# 4‐Thiazolidinone/2,4‐Thiazolidinedione–Sulfonamide Hybrids: A Concise Review of Their Design and Pharmacological Significance

**DOI:** 10.1002/cmdc.70557

**Published:** 2026-10-28

**Authors:** Subham Kumar Vishwakarma, Andressa Francielli Bonjorno, Sukhwant Singh, Christian S. Carnero Canales, Jean Leandro dos Santos, Cesar Augusto Roque‐Borda, Fernando Rogério Pavan

**Affiliations:** ^1^ Tuberculosis Research Laboratory School of Pharmaceutical Sciences São Paulo State University (UNESP) Araraquara Brazil; ^2^ Laboratory of Drug Planning and Development School of Pharmaceutical Sciences São Paulo State University (UNESP) Araraquara Brazil; ^3^ Sage Institute of Research and Technology‐Pharmacy Sanjeev Agrawal Global Educational University (SAGE) Bhopal India; ^4^ Vicerrectorado de Investigación Universidad Católica de Santa María Arequipa Peru

**Keywords:** hybridization, medicinal chemistry, multitarget drug design, sulfonamide, sulfonamide–thiazolidinone hybrids, thiazolidinedione

## Abstract

Molecular hybridization represents a promising strategy in drug‐design and the discovery of novel biologically active molecules, as it can enhance molecular affinity and potency while simultaneously reducing resistance and side effects. This review explores the recent advancements in the design, synthesis, and biological evaluation of hybrid molecules that combine sulfonamide with thiazolidinone or thiazolidine‐2,4‐dione scaffolds. The review covered studies published from 2009 to 2025 and focused exclusively on compounds containing both a sulfonamide moiety and a thiazolidinone or thiazolidinedione framework in the same molecule. These hybrid compounds have attracted considerable attention due to their potential to address the complexity of multifactorial diseases such as cancer, infections, and metabolic disorders, which often involve multiple biological pathways. By integrating features from both sulfonamides and thiazolidines, researchers have developed molecules with enhanced pharmacological profiles, including improved antimicrobial, anticancer, anti‐inflammatory, antidiabetic, and anticonvulsant activities. The review highlights how these hybrids often display multitarget effects, making them promising candidates for overcoming limitations of traditional single‐target drugs, such as resistance and limited efficacy. Key findings from recent studies are summarized, with a focus on the structural aspects that contribute to biological activity. Sulfonamide–thiazolidinone and sulfonamide–thiazolidinedione hybrids represent a versatile and valuable strategy in the search for new treatments for complex diseases.

## Graphical abstract

Sulfonamide–thiazolidinone hybrids integrate complementary pharmacophores to generate multifunctional scaffolds with broad pharmacological potential. Their structural versatility supports antimicrobial, antifungal, antioxidant, anticancer, anti‐inflammatory, antidiabetic, anticonvulsant, and enzyme‐inhibitory activities. Rational hybridization emerges as a valuable strategy to enhance biological efficacy, selectivity, and multitarget activity while expanding opportunities for the design of therapeutically relevant heterocyclic compounds.

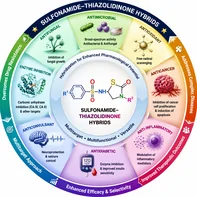

## 1 Introduction

The increasing prevalence of multifactorial diseases, including cancer, infectious, inflammatory, and metabolic disorders, presents a significant challenge for contemporary drug discovery efforts [1]. This complexity arises from the involvement of multiple dysregulated biological pathways, which restricts the efficacy of conventional single‐target therapies. Consequently, multitarget strategies are being developed to address these limitations [2, 3]. There is growing interest in multitarget ligands capable of modulating several biological targets simultaneously, an approach that may reduce the development of drug resistance [4, 5]. Heterocyclic compounds serve as essential scaffolds in medicinal chemistry due to their structural diversity and broad spectrum of biological activities [6, 7], making them widely uses in drug development. Molecular hybridization, which integrates distinct pharmacophoric units within a single chemical entity, has emerged as a prominent strategy in multitarget drug design [8]. Sulfonamide and thiazolidine‐derived pharmacophores exhibit diverse pharmacological activities [6, 9], and their combination in hybrid structures offers a promising avenue for generating compounds with enhanced biological and pharmacokinetic properties.

Heterocycles, which represent the most prevalent class in traditional organic synthesis, exemplify this overlap. Numerous biologically active compounds containing heteroatoms such as nitrogen, sulfur, and oxygen have long attracted research interest. Thiazolidine is a saturated five‐membered heterocyclic system containing sulfur and nitrogen atoms at positions 1 and 3, respectively. Among its carbonyl‐containing derivatives, thiazolidin‐4‐one and thiazolidine‐2,4‐dione represent two important but structurally distinct scaffolds in medicinal chemistry [10, 11]. Thiazolidin‐4‐one contains a single carbonyl group at the C‐4 position, whereas thiazolidine‐2,4‐dione contains two carbonyl groups at the C‐2 and C‐4 positions (Figure 1a) [12]. Although both scaffolds share the 1,3‐thiazolidine scaffold, the presence of the additional carbonyl group in thiazolidine‐2,4‐dione confers distinct chemical and physicochemical properties. Accordingly, thiazolidin‐4‐one and thiazolidine‐2,4‐dione derivatives are treated as two separate structural classes throughout this review [13, 14].

**FIGURE 1:**
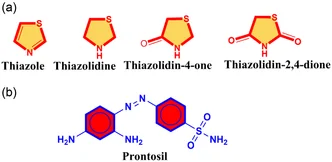
(a) Chemical structures of thiazole, thiazolidine, thiazolidinone, and thiazolidinedione pharmacophore. (b) Structure of the first sulfonamide drug, Prontosil.

Derivatives, containing both sulfur and nitrogen, have fueled research due to their potential biological applications in regulating various physiological processes [15, 16, 17]. Derivatives based on these two heterocyclic scaffolds have been extensively investigated due to their diverse pharmacological properties, including antihyperglycemic [6, 18, 19], antioxidant [20, 21], anticancer [6, ‍22, 23], anti‐inflammatory [24, 25], anti‐arthritic [26], anti‐obesity [27], anti‐microbial [28, 29, 30, 31], and anticonvulsant [32, 33]. One effect that has been studied extensively in thiazolidinediones derivatives is their ability to lower blood sugar. This effect has also been used to make glitazone drugs like rosiglitazone [34], pioglitazone [35, 36], troglitazone [37], and lobeglitazone [38] (Figure 2).

**FIGURE 2:**
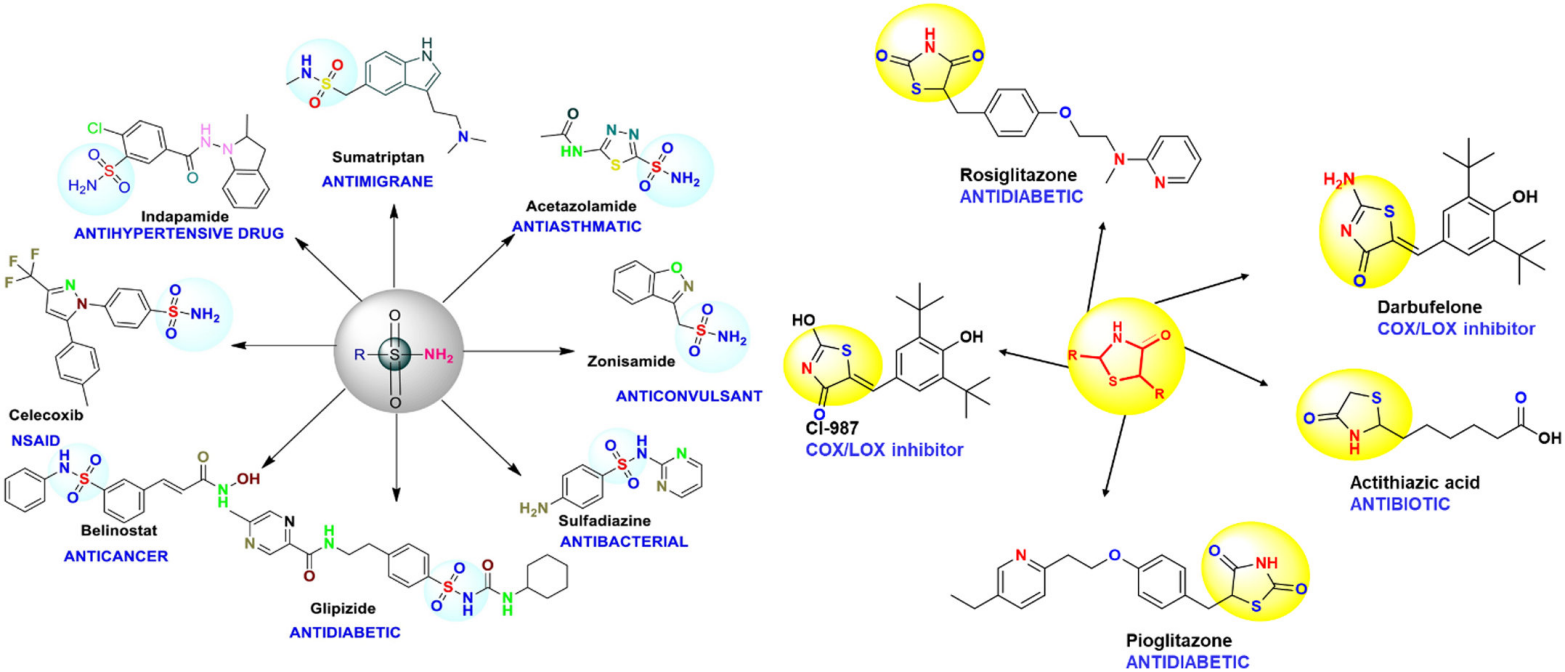
FDA‐approved drugs containing sulfonamide and thiazolidinone/thiazolidinedione scaffolds as privileged pharmacophores, recreated with permission from [39]. Copyright 2025, Elsevier B.V.

Sulfonamides represent an essential class of compounds in synthetic and medicinal chemistry and are sulfur‐containing derivatives of sulfanilamide. Sulfonamide structures are organo‐sulfur compounds containing the ―SO_2_NH_2_ and/or ―SO_2_NH― group and are characteristic of the existence of sulfanilamide group and distinct six‐ or five‐membered heterocyclic rings [40]. These antimicrobial agents, which are highly effective against pyogenic bacterial infections, are classified as sulfa drugs. The discovery of their therapeutic potential dates to 1932, when German bacteriologist Gerhard Domagk identified the antimicrobial properties of prontosil red (Figure 1b), a dye used to treat microbial infections in animals [41, 42, 43]. In recent years, sulfa drugs have been used to treat bacterial infections that are resisted to other antibiotics, as well as conditions like acne and urinary tract infections [17, 41]. Sulfonamides exhibit a broad spectrum of biological activities including antibacterial [17, 44, 45], antifungal [45, 46, 47], anti‐inflammatory [48, 49, 50], antiprotozoal [51, 52], anticancer [53, 54, 55, 56], carbonic anhydrase (CA) inhibitor [55, 57, 58, 59, 60], antidiabetic [61, 62, 63], and diuretic [64, 65, 66]. Along with treating obesity [67], rheumatoid arthritis [68], ulcerative colitis [49], scalds [69], male erectile dysfunction, and phosphodiesterase‐5 inhibitors like sildenafil [70], also effective in treating infection of the eyes, intestines, and urinary system [71, 72, 73] (Figure 2).

Hybridization of sulfonamide and thiazolidin‐4‐one or thiazolidine‐2,4‐dione moieties represents a strategic drug‐design approach aimed at combining the beneficial attributes of each class into a single molecular entity. This strategy has the potential to enhance biological activity, improve pharmacokinetic profiles, and reduce the likelihood of resistance development. Hybrid compounds often exhibit more than one mode of action, targeting multiple biological pathways simultaneously, which can be particularly advantageous for treating complex diseases and infections. This review summarizes recent progress in the design, synthesis, and biological evaluation of sulfonamide–thiazolidin‐4‐one and sulfonamide–thiazolidine‐2,4‐dione hybrids, with an emphasis on their multitarget potential in infectious and other diseases.

## 2 Pharmacophoric Roles and Mechanisms of Action of Sulfonamide and Thiazolidine‐Derived Scaffolds

Sulfonamides are a chemically versatile drug class whose sulfonamide functionality can participate in distinct target‐dependent interactions. Two well‐established mechanistic examples include: (1) structural mimicry and competitive antagonism of *p*‐aminobenzoic acid (PABA) by classical antibacterial sulfonamides at bacterial dihydropteroate synthase (DHPS), thereby disrupting folate biosynthesis, and (2) coordination of the deprotonated nitrogen of primary sulfonamides to active‐site Zn^2+^ in metalloenzymes, particularly carbonic anhydrases, resulting in enzyme inhibition (Figure 3) [40, 74]. These mechanisms depend on the specific sulfonamide structure and biological target and therefore do not apply to all sulfonamide‐containing compounds.

**FIGURE 3:**
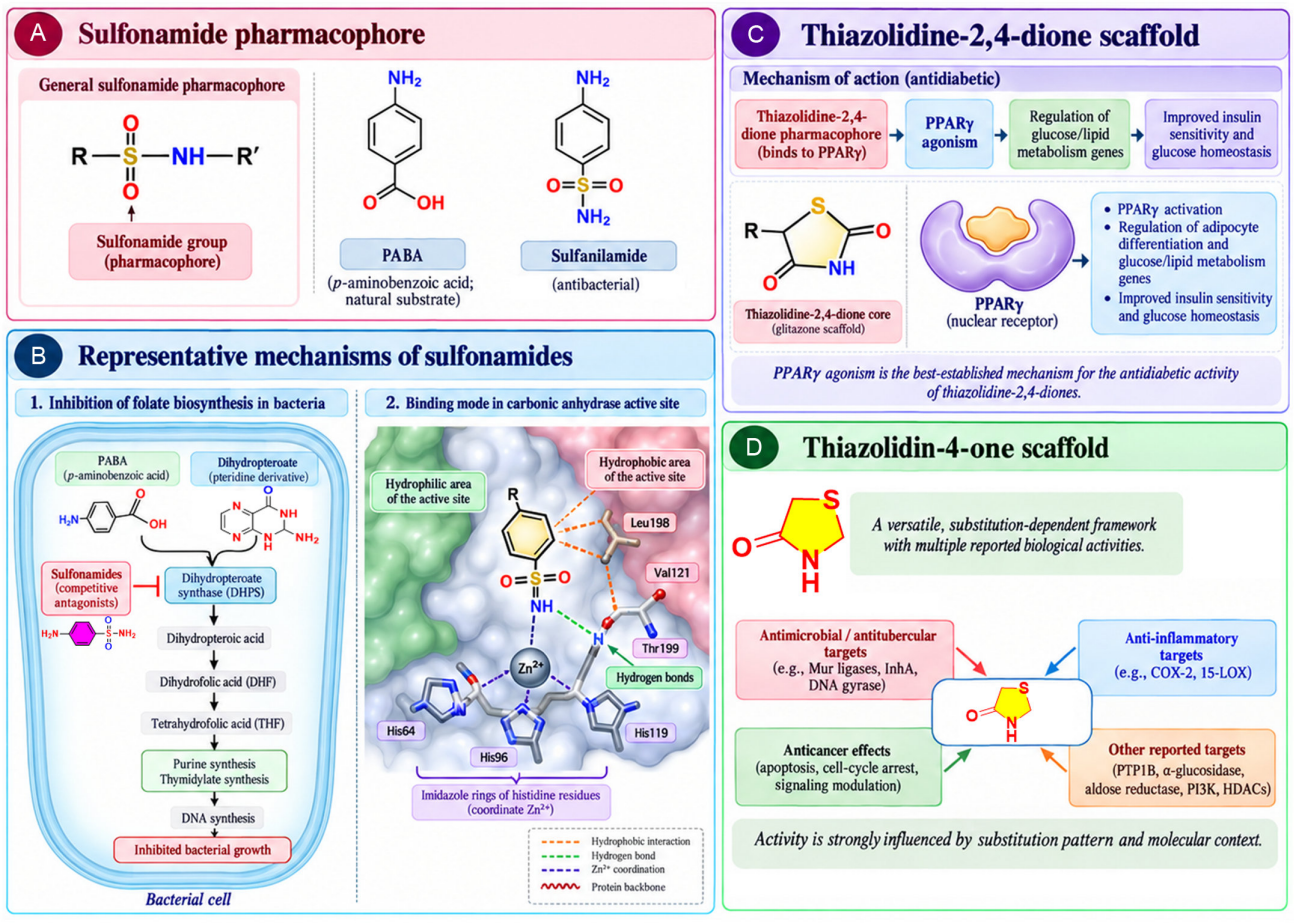
Pharmacophoric roles and mechanisms of action of sulfonamide and thiazolidine‐derived scaffolds. (A) General sulfonamide structure and structural relationship between PABA and sulfanilamide. (B) Sulfonamide‐mediated DHPS inhibition in bacterial folate biosynthesis and Zn^2+^ coordination in carbonic anhydrase. (C) Thiazolidine‐2,4‐dione‐mediated PPAR‐γ agonism and its role in glucose and lipid homeostasis. (D) Target‐dependent pharmacological roles of thiazolidin‐4‐one derivatives.

Thiazolidin‐4‐one and thiazolidine‐2,4‐dione are significant heterocyclic scaffolds associated with a wide range of biological activities [75]. The best‐characterized mechanism involves thiazolidine‐2,4‐dione‐based antidiabetic agents, such as rosiglitazone and pioglitazone, which activate peroxisome proliferator‐activated receptor‐γ (PPAR‐γ) and thereby modulating genes involved in glucose and lipid metabolism and improving insulin sensitivity and glucose homeostasis (Figure 3) [76, 77, 78]. Beyond this established mechanism, thiazolidine‐4‐one and thiazolidine‐2,4‐dione derivatives have been reported to interact with various other biological targets [79]. Selected derivatives exhibit antimicrobial and antitubercular activity by inhibiting enzymes essential to bacterial processes, including Mur ligases, InhA, and DNA gyrase [80, 81] . Anti‐inflammatory effects have been linked to PPAR‐γ activation as well as inhibition of inflammatory mediators and enzymes such as COX‐2 and 15‐LOX [82]. In anticancer studies, both PPAR‐γ‐dependent and PPAR‐γ‐independent mechanisms have been reported, including apoptosis induction, cell‐cycle arrest, and modulation signaling pathways involved in cell proliferation and survival [83, 84, 85]. Additionally, other derivatives have been shown to inhibit aldose reductase, PTP1B, α‐glucosidase, PI3K, and HDACs [86, 87]. Thus, the pharmacological effects of these scaffolds are strongly dependent on substitution pattern, molecular context, and target and cannot be explained by a single mechanism of action.

## 3 Biological Activity of 4‐Thiazolidinones, 2,4‐ Thiazolidinediones, and Sulfonamides Hybrids

### 3.1 Antimicrobial Activity

Shorey et al. synthesized sulfamerazine–indolo‐thiazolidinone hybrids using a microwave‐assisted, environmentally benign cyclocondensation approach (Figure 4) [88]. Among the synthesized derivatives, compounds 1 and 2 showed notable antibacterial activity against *Escherichia coli* and *Bacillus subtilis*. Compound 1 exhibited the highest activity against *E. coli*, with a zone of inhibition of 15.2 mm at 400 µg/mL, corresponding to 57.59% of the activity of ciprofloxacin. Compound 2 was the most active against *B. subtilis*, produced an inhibition zone of 15 mm and showing 83.33% activity relative to the standard drug. In contrast, compound 3 displayed the most favorable antifungal activity, producing inhibition zones of 22 mm against *A* *spergillus niger* and 28 mm against *A* *spergillus flavus* at the same concentration, corresponding to 78.57% and 93.33% of the activity of fluconazole, respectively. These findings identify compound 2 as the most active antibacterial derivative and compound 3 as the most active antifungal as well as the most promising broad‐spectrum compound of the series.

**FIGURE 4:**
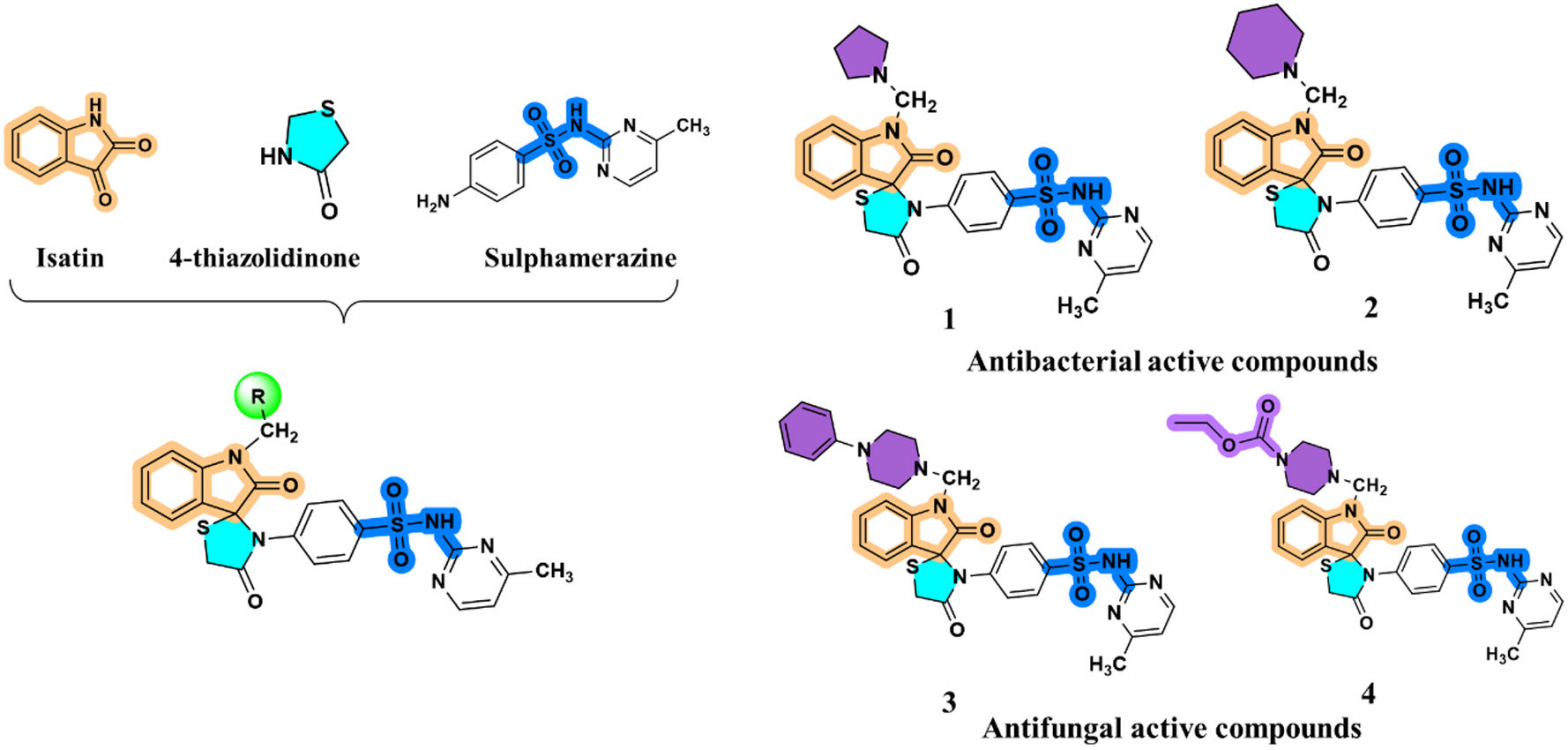
Design strategy for synthesizing hybrids of thiazolidin‐4‐one–sulfonamide and structures of compounds showing antimicrobial activity.

Alagawadi and Alegaon reported the synthesis of novel 2,4‐thiazolidinedione–sulfonamide hybrids using 5‐aminothiadiazole sulfonamide via Knoevenagel condensation (Figure 5) [89]. Among the tested derivatives, compounds 5 and 7 showed the most promising antibacterial activity [90]. Compound 5 exhibited MIC values of 128, 32, 8, and 4 µg/mL against *E. coli*, *Pseudomonas aeruginosa*, *Staphylococcus aureus*, and *Enterococcus faecalis*, respectively, whereas compound 7 showed MIC values of 64, 64, 8, and 4 µg/mL against the same strains. In comparison, the standard drug ampicillin displayed MIC values of 2, 2, 1, and 2 µg/mL, respectively. Although both compounds were less active than ampicillin, their comparatively low MIC values, particularly against *S. aureus* and *E. faecalis*, identified them as the most active antibacterial compounds of the series [89]. In the antifungal assay, compound 5 exhibited the most pronounced activity, with MIC values of 1, 2, 4, and 4 µg/mL against *Candida* *albicans*, *Cryptococcus* *neoformans* *neoformans*, *A. flavus*, and *A. niger*, respectively. The standard antifungal drug ketoconazole showed corresponding MIC values of 2, 1, 2, and 1 µg/mL. Notably, compound 5 was more active than ketoconazole against *C. albicans*, with an MIC of 1 µg/mL compared with 2 µg/mL for the standard, while showing lower activity against the other fungal strains. Compound 6 also displayed notable antifungal activity, with MIC values of 4, 8, 4, and 4 µg/mL, respectively. Overall, compound 5 emerged as the most promising antimicrobial derivative of the series.

**FIGURE 5:**
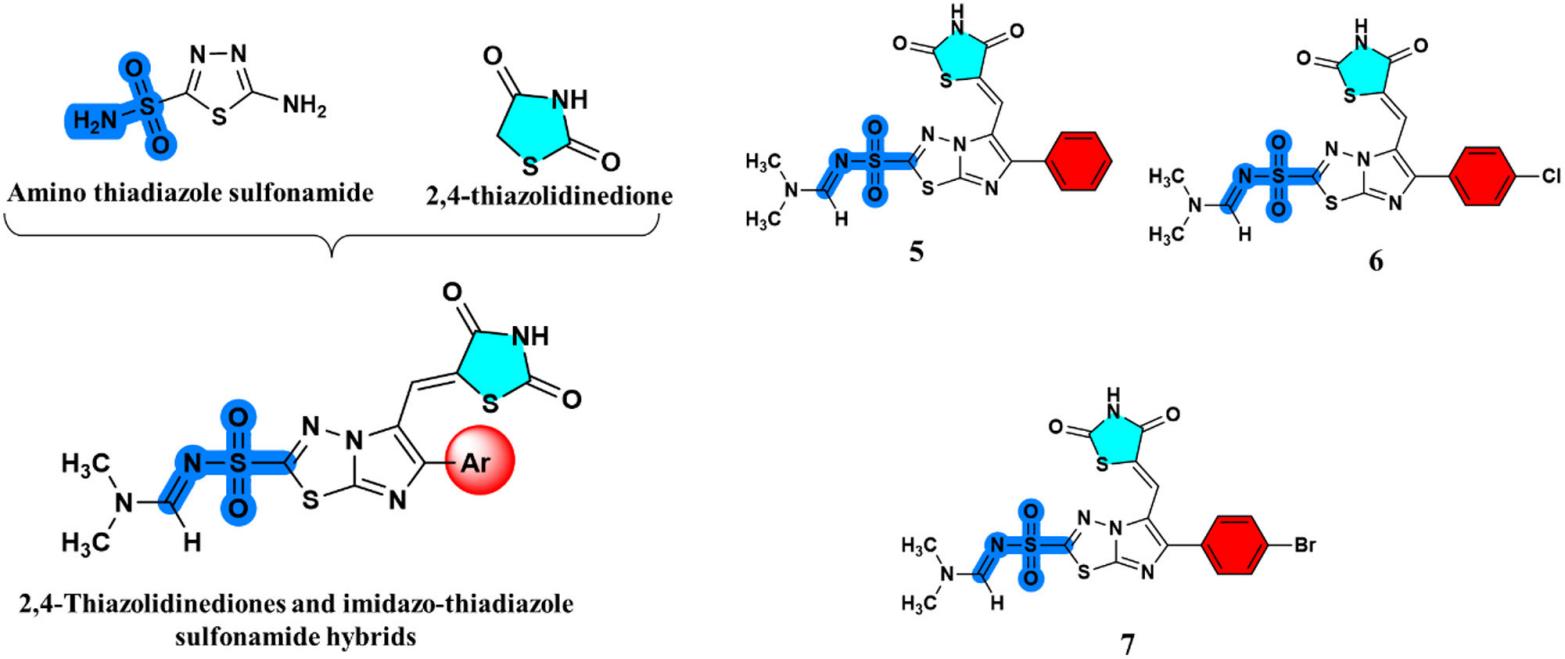
Hybridization strategy of sulfonamide–thiazolidine‐2,4‐dione derivatives and structures of the active compounds against tested *bacterial and fungal* strains.

Desai et al. reported a novel series of 4‐thiazolidinones containing sulfonamide and synthesized via Schiff base cyclocondensation with thioglycolic acid (Figure 6) [29]. The antimicrobial activity of compounds was evaluated against bacterial strains, including *E. coli* and *B. cirroflagellosus*, as well as fungal species *A. niger* and *Colletotrichum capsici*. Compound 8 showed the highest antibacterial activity against *E. coli*, achieving 100% relative inhibition, equivalent to the standard drug cotrimoxazole. In contrast, the compounds exhibited only weak activity against *B. cirroflagellosus*, with inhibition values generally between 15% and 30% of the standard. Regarding antifungal activity, compound 9 achieved 78.7% inhibition against *A. niger* and 206.2% inhibition against *C. capsici* relative to fluconazole. Compound 10 exhibited the highest activity against *C. capsici*, with 299.39% relative inhibition, surpassing fluconazole (100%).

**FIGURE 6:**
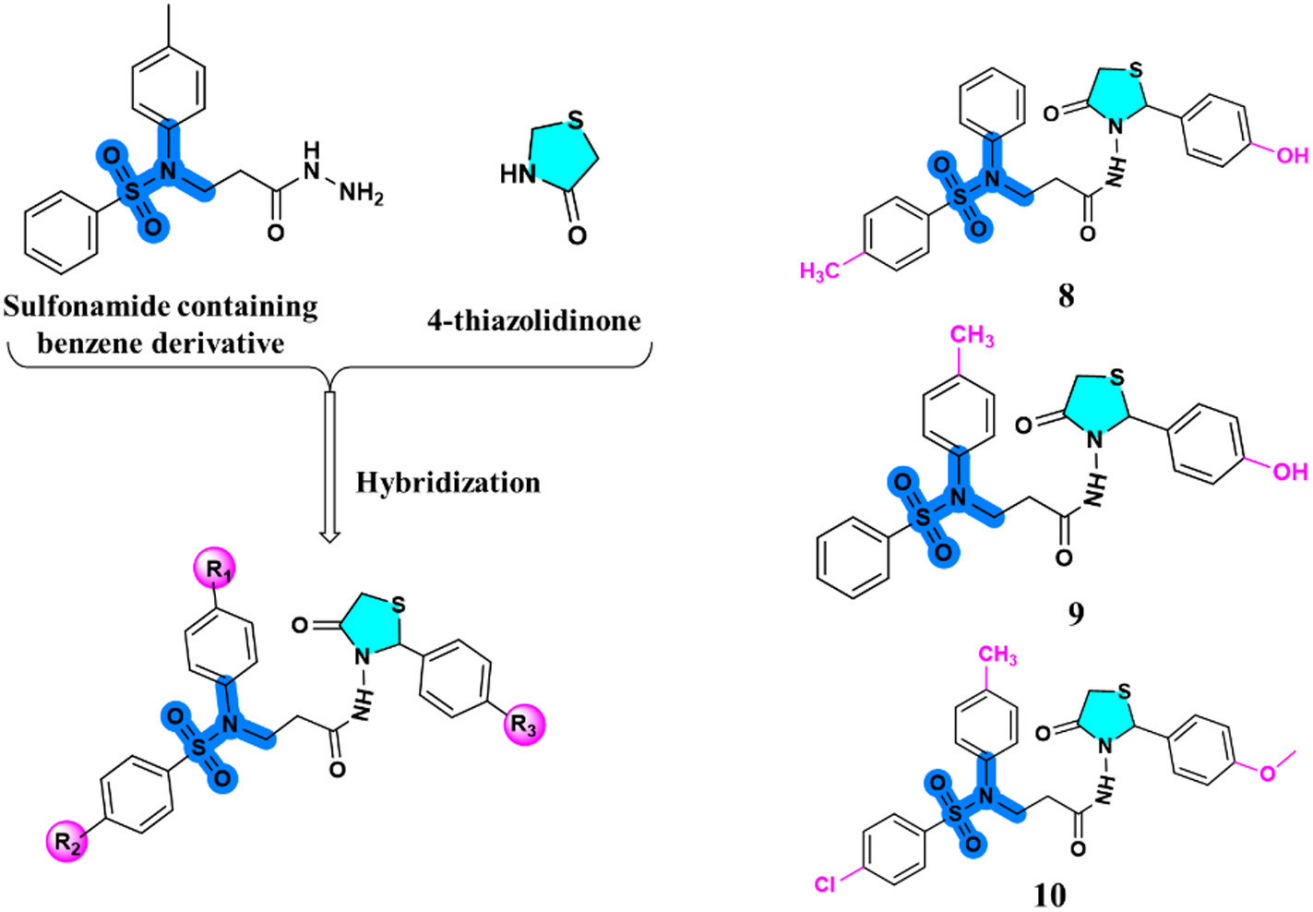
Hybridization strategy for the synthesis of sulfonamide‐containing 4‐thiazolidinones and derivatives identified in the antibacterial and antifungal assay.

Subhedar *et* al. designed and synthesized a series of N‐sulfonamide‐thiazolidin‐4‐one hybrids fused with sulfonyl hydrazide moieties via a solvent‐free reaction (Figure 7) [91]. Among the synthesized thiazolidin‐4‐one derivatives, compounds 11 and 12 showed the most notable antifungal activity. Compound 11 exhibited MIC values of 25 µg/mL against *C. albicans*, *A. flavus*, and *A. niger*, which were equal to miconazole (25 µg/mL) against these strains. Compound 12 also showed MIC values of 25 µg/mL *against C*. *albicans*, *F. oxysporum*, and *A. flavus*, comparable to miconazole (25 µg/mL). However, both compounds were less active than fluconazole, which showed MIC values of 6.25 µg/mL against *C. albicans*, *F. oxysporum*, *and A. flavus* and 12.5 µg/mL against *A. niger*. In the DPPH radical scavenging assay, compound 13 exhibited the highest antioxidant activity, with an IC_50_ value of 16.25 µg/mL, slightly lower than that of the standard antioxidant BHT (16.47 µg/mL), indicating comparable and marginally higher radical‐scavenging activity. Molecular docking studies further corelated their biological relevance by revealing favorable binding energies (−7.04 to −7.31 kcal/mol) and strong interactions with the target enzyme sterol 14α‐demethylase (CYP51).

**FIGURE 7:**
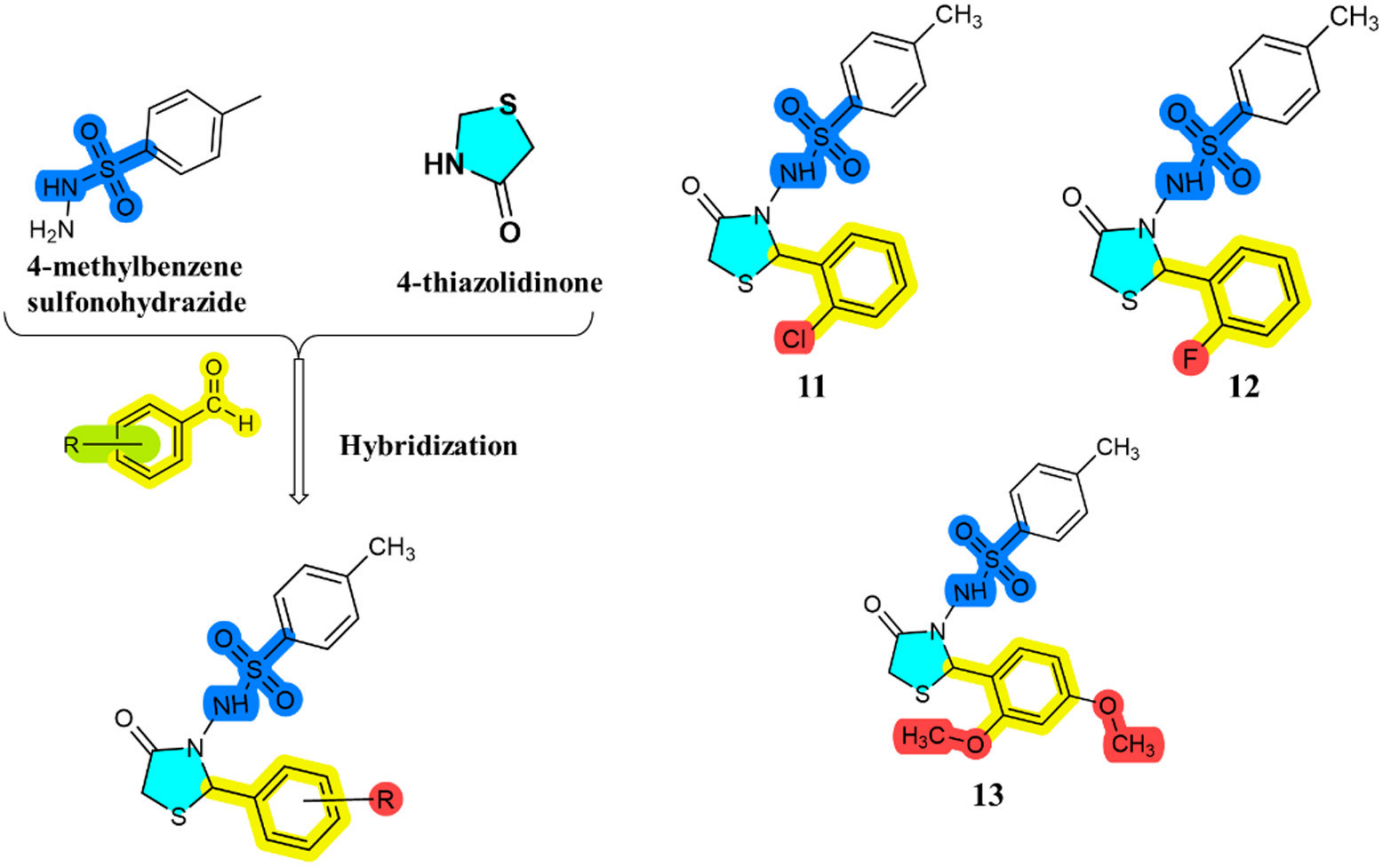
Hybridization strategy for the synthesis of novel 4‐thiazolidinone–sulfonamide derivatives and structures of the most promising compounds identified from the series.

Bhat et al. designed and synthesized novel sulfamethoxazole–4‐thiazolidinone hybrids and evaluated their antimycobacterial activity against *Mycobacterium bovis BCG* and *M. tuberculosis H37Ra* (Figure 8) [92]. Among the synthesized derivatives, compound 14 showed the highest activity, with IC_50_ and IC_90_ values of 0.016 and 0.058 µg/mL against *M. bovis* BCG and 0.07 and 0.43 µg/mL against *M. tuberculosis H37Ra*, respectively. Compound 15 also showed pronounced activity, with IC_50_/IC_90_ values of 0.02/0.13 µg/mL against *M. bovis BCG* and 0.12/5.31 µg/mL against *M. tuberculosis H37Ra*. Rifampicin, used as the reference drug, showed IC_50_/IC_90_ values of 0.0043/0.0173 µg/mL and 0.0019/0.020 µg/mL against the respective strains. Although the synthesized derivatives were less potent than rifampicin, compounds 14 and 15 showed marked antimycobacterial activity. The most active derivatives were further evaluated for cytotoxicity against MCF‐7, HCT 116, and A549 cell lines and showed GI_50_ and GI_90_ values >100 µg/mL, indicating low cytotoxicity at the tested concentrations.

**FIGURE 8:**
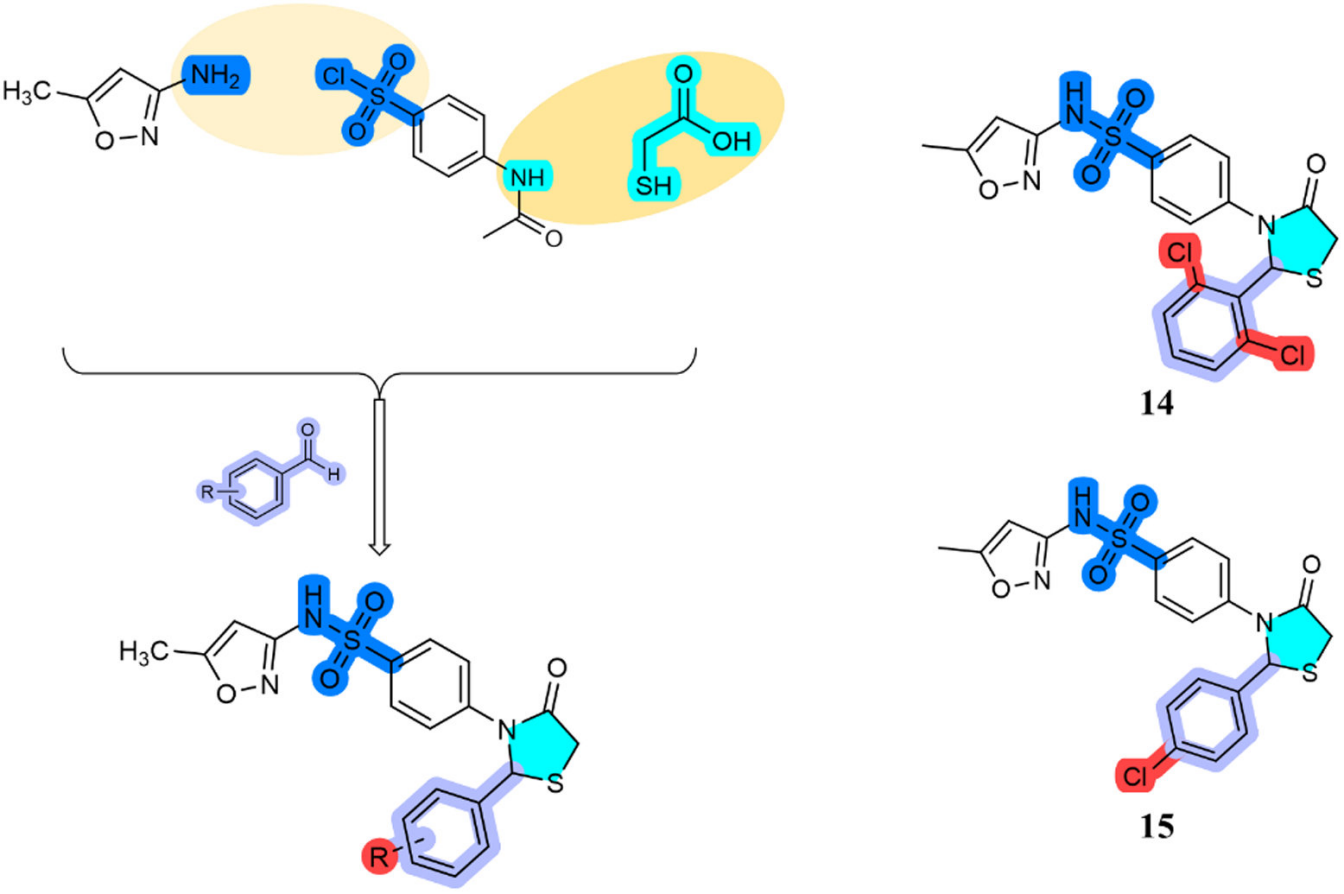
Design of sulfamethoxazole–4‐thiazolidinone hybrids and structures of the most active antimycobacterial derivatives 14 and 15 within the series.

Murugesan et al. developed sulfonamide‐containing thiazolidin‐4‐one derivatives via a one pot reaction (Figure 9) [93]. The synthesized compounds were evaluated against the *X4‐tropic HIV‐1*_*IIIB*_ and *R5‐tropic HIV‐1*_*ADA5*_ strains and for inhibition of HIV‐1 reverse transcriptase (RT). Compound 16 showed EC_50_ values of 29.40 µM against *HIV‐1*_*IIIB*_ and 4.55 µM against HIV‐1_ADA5_, with corresponding selectivity indices of 2.57 and 16.63, respectively, and produced 57.9% inhibition of HIV‐1 RT at 100 µg/mL. Compound 17 exhibited moderate activity against both strains (EC_50_: 63.11 µM for HIV‐1_IIIB_ and 64.6 µM for HIV‍‐‍1_ADA5_) with selectivity indices of 2.15 and 2.10, respectively, and showed 56.3% HIV‐RT inhibition. In comparison, the reference drug nevirapine displayed an EC_50_ of 0.03 µM against both HIV‍‐‍1 strains, selectivity indices of 2057, and 99.20% inhibition of HIV‐1 RT. Thus, compound 16 showed greater antiviral activity than compound 17, particularly against the HIV‐1_ADA5_ strain, although both compounds were markedly less potent and less selective than nevirapine. These findings indicate measurable anti‐HIV activity for compounds 16 and 17 and support further structural optimization of this sulfonamide—thiazolidin‐4‐one hybrid.

**FIGURE 9:**
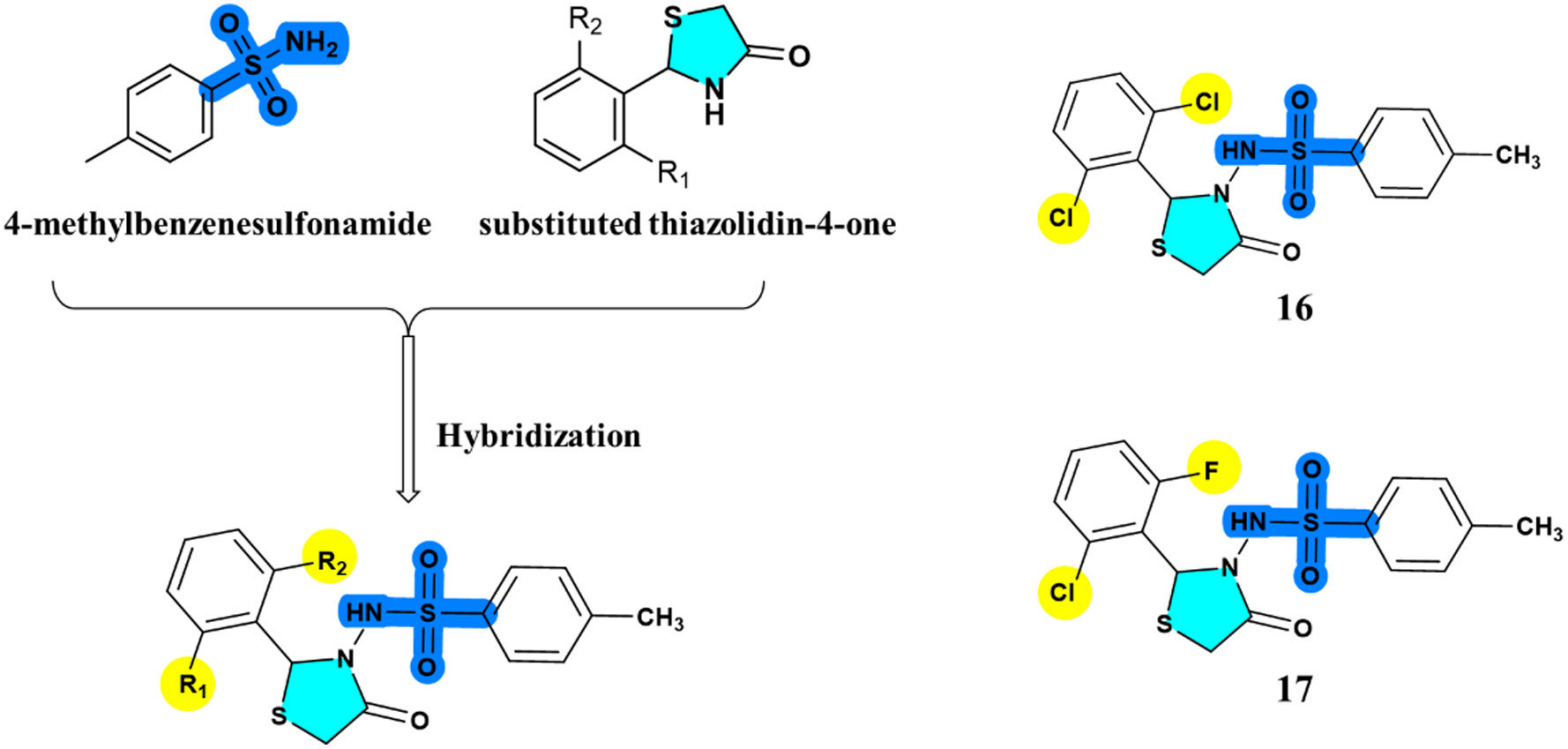
Hybridization strategy for the design of sulfonamide‐containing thiazolidin‐4‐one derivatives and structures of the selected anti‐HIV compounds 10 and 11.

### 3.2 Anticancer Activity

Hafez et al. reported the synthesis of novel triazolo[4,3‐‍a]pyrimidin‐6‐sulfonamides bearing thiazolidinone derivatives using chlorosulfonic acid in thionyl chloride (Figure 10) [94]. The hybrid compounds combine triazolopyrimidine, sulfonamide, and thiazolidinone pharmacophores. Following preliminary screening at 10^−4^ M, the most active derivatives were further evaluated against the NCI panel of 60 human cancer cell lines. Compound 18 showed broad antiproliferative activity, with GI_50_ values ranging from 5.89 × 10^−6^ to 3.71 × 10^−5^ M, and produced total growth inhibition in 56 cell lines at concentrations of 2.1–8.94 × 10^−5^ M. It also showed cytotoxic activity against 31 cell lines, with notable selectivity toward renal and prostate cancer cells. Compound 19 completely inhibited the growth of 20 cell lines and showed marked activity against the leukemia panel, whereas compound 20 displayed cytotoxicity against four leukemia cell lines, with LC_50_ values ranging from 7.15 × 10^−5^ to 8.68 × 10^−5^ M. Compound 21 produced 50% growth inhibition in 26 cell lines and total growth inhibition in 24 cell lines, with pronounced activity against the leukemia SR cell line (TGI = 3.18 × 10^−6^ M; LC_50_ = 9.20 × 10^−6^ M) and cytotoxicity against the HCC‐2998 colon cancer cell line (LC_50_ = 6.48 × 10^−5^ M). Academia The structure–activity relationship indicated that substitution at the 6‐position of the triazolopyrimidine sulfonamide scaffold strongly influenced activity, with thienyl‐substituted thiazolidinone derivatives bearing 4‐methylpiperazine or morpholine showing the most favorable antitumor profiles.

**FIGURE 10:**
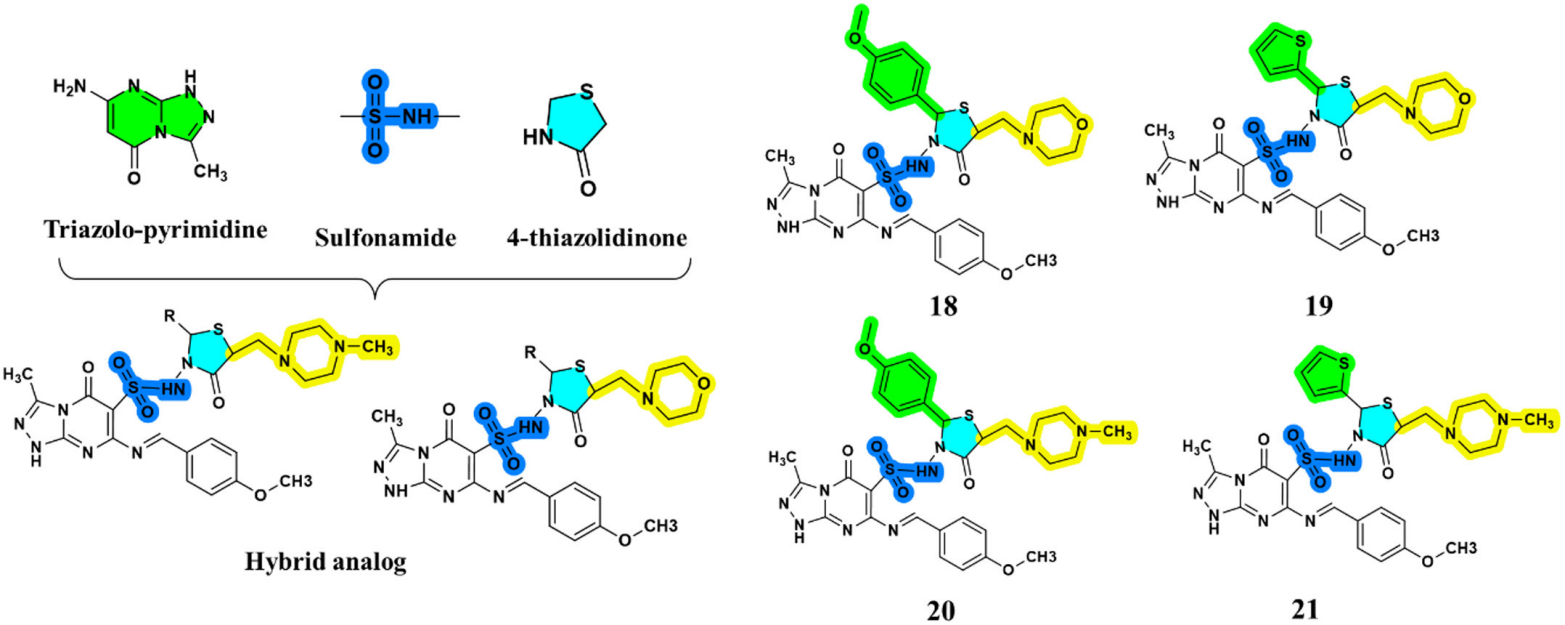
Design strategy of hybrids with most favorable compound with antitumor activity.

Kamel et al. synthesized novel hybrids of sulphapyridine and 2‐arylthiazolidin‐4‐one (Figure 11) [23]. The synthesized compounds were evaluated for anticancer activity against MCF7 breast carcinoma and HeLa cervical carcinoma cell lines. Among the tested derivatives, compound 22 showed the highest activity, with IC_50_ values of 1.07 µg/mL against MCF7 and 1.95 µg/mL against HeLa cells. These values were considerably lower than those of doxorubicin (6.71 and 8.72 µg/mL, respectively), indicating greater activity than doxorubicin under the same assay conditions. However, compound 22 was less active than 5‐fluorouracil, which showed IC_50_ values of 0.67 µg/mL against MCF7 and 1.01 µg/mL against HeLa. Compound 23 exhibited IC_50_ values of 2.68 µg/mL against MCF7 and 2.01 µg/mL against HeLa. Although compound 23 was also more active than doxorubicin, it was approximately fourfold less active than 5‐flurouracil against MCF7 cells and about two‐fold less active against HeLa cells.

**FIGURE 11:**
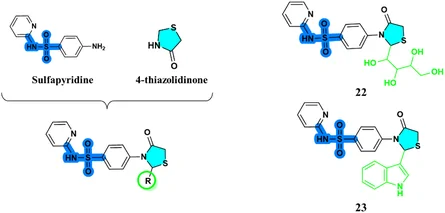
Hybridization strategy for the design of sulfapyridine–thiazolidin‐4‐one hybrids with anticancer activity.

Kurt et al. synthesized a series of benzenesulfonamide–thiazolidinone hybrids to investigate their cytotoxic activity against DLD‐1, HT‐29, and COLO‐205 colorectal cancer cell lines (Figure 12) [95]. Compound 24 showed the highest activity against DLD‐1 cells with IC_50_ of 25.82 μM, whereas compounds 25 and 26 exhibited the strongest activity against COLO‐205 and HT‐29 cells with IC_50_ of 30.99 and 114.62 μM, respectively. In comparison, the reference drug 5‐fluorouracil showed IC_50_ values of 4.28, 17.17, and 18.31 µM against DLD‐1, HT‐29, and COLO‐205 cells, respectively, indicating greater cytotoxic potency than the synthesized derivatives. Compound 27 showed an IC_50_ value of 35.93 µM against DLD‐1 cells and displayed the highest selectivity index (SI = 29.9) of the series, compared with an SI of 2.8 for 5‐FU. It also showed markedly lower cytotoxicity toward normal CCD‐986Sk cells than 5‐FU. Apoptosis studies further showed that compound 27 at 30 µM produced an apoptotic profile similar to that observed with 5‐FU at 5 µM, suggesting its favorable selectivity toward colorectal cancer cells.

**FIGURE 12:**
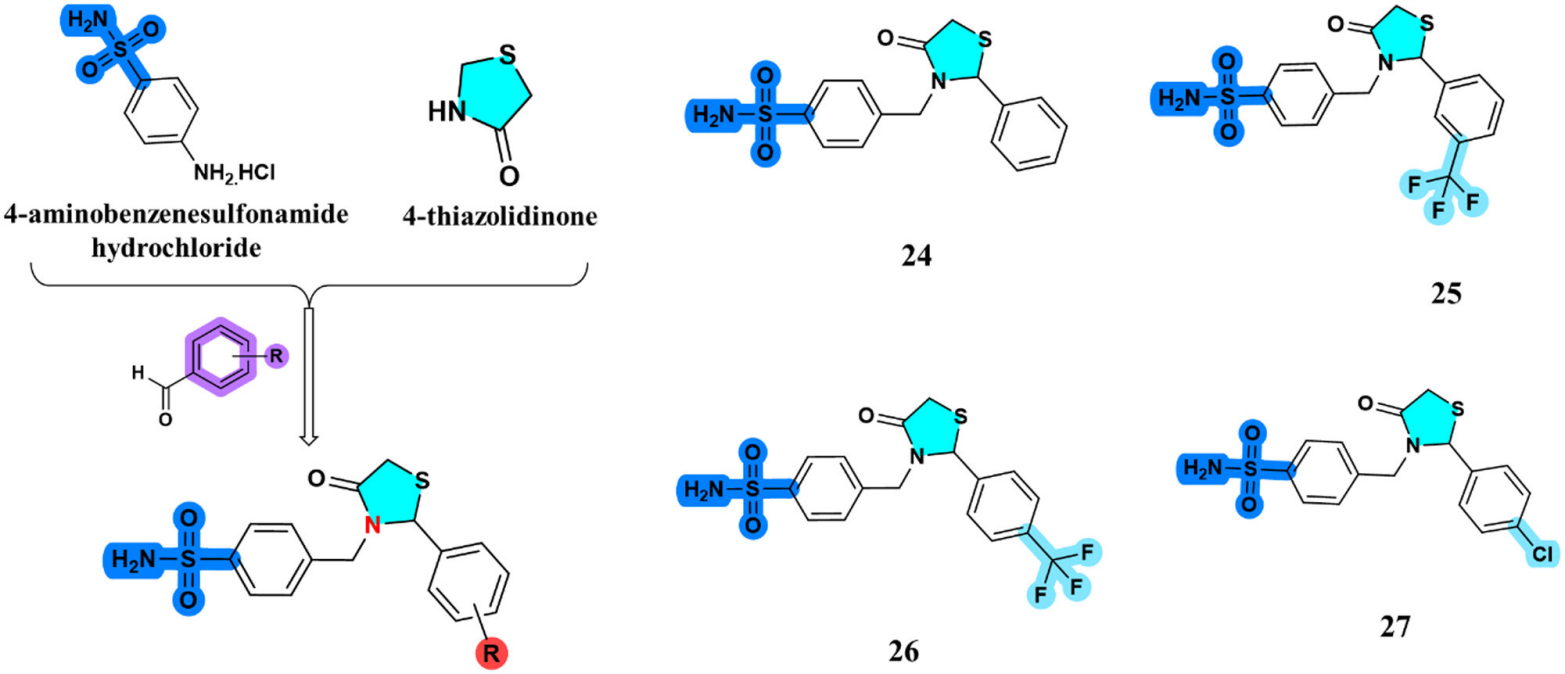
Design strategy of sulfonamide and 4‐thiazolidinone hybridization and active anticancer compounds from series.

### 3.3 Anti‐inflammatory Activity

Bayoumi et al. designed and synthesized hybrids of thiazolidin‐2,4‐dione and 4‐aminobenzenesulfonamide derivatives (Figure 13) [96]. Among these hybrids, Compound 28 showed the highest anti‐inflammatory activity, producing a 48.75 ± 7.11% increase in paw thickness and 58.4% inhibition of edema at 100 mg/kg, indicating strong anti‐inflammatory efficacy. Compound 29 also displayed considerable activity, with a 62.3 ± 4.77% increase in paw thickness and 46.8% inhibition of edema at 100 mg/kg, suggesting a moderate yet meaningful anti‐inflammatory effect. In comparison, the standard drug diclofenac K (10 mg/kg) produced a 32.48 ± 3.24% increase in paw thickness and 72.29% inhibition of edema, indicating that both compounds were less active than the standard under the assay conditions. Nevertheless, compound 28 emerged as the most active derivative of the series in the carrageenan‐induced rat paw edema model, supporting its potential for further optimization as an anti‐inflammatory agent.

**FIGURE 13:**
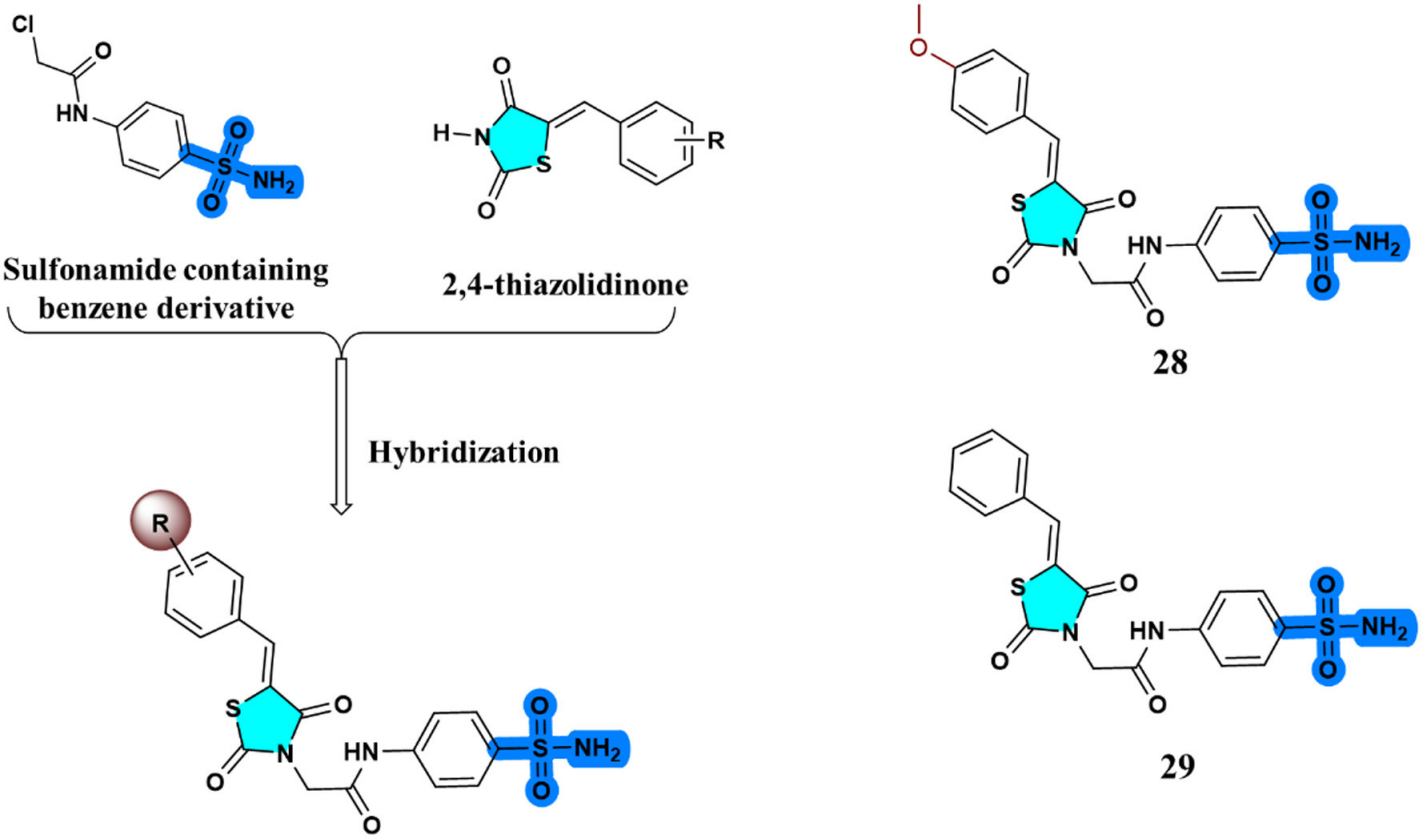
Hybridization strategy for the design of thiazolidine‐2,4‐dione—sulfonamide hybrids and structures of the anti‐inflammatory derivatives, compounds 28 and 29.

Ashour et al. designed and synthesized new hybrids combining pyrazolyl benzenesulfonamide with polysubstituted 4‐thiazolidinone (Figure 14) [97]. Among the tested compounds, compounds 30 and 31 showed the most pronounced anti‐inflammatory activity. Compound 30 produced edema volumes of 0.47 ± 0.01, 0.49 ± 0.01, and 0.54 ± 0.03 cm^3^ at 1, 2, and 4 h, corresponding to 40, 45, and 56% inhibition, respectively, with an ED_50_ value of 14.54 mg/kg. Compound 31 showed edema volumes of 0.47 0.03, 0.49 ± 0.02, and 0.55 ± 0.01 cm^3^, corresponding to 40, 45, and 54% inhibition, with an ED_50_ of 15.13 mg/kg. In comparison, the standard drug diclofenac sodium produced 36, 38, and 58% inhibition at 1, 2, and 4 h, respectively, with an ED_50_ of 13.95 mg/kg. Thus, compounds 30 and 31 showed slightly greater inhibition than diclofenac during the first 2 h, whereas diclofenac produced slightly higher inhibition after 4 h and showed a marginally lower ED_50_. Compound 30 also inhibited COX‐1 and COX‐2 with IC_50_ values of 4.50 and 1.06 µM, respectively, while compound 31 showed corresponding IC_50_ values of 28.50 and 8.98 µM, with COX‐2 selectivity indices of 4.24 and 3.17, respectively.

**FIGURE 14:**
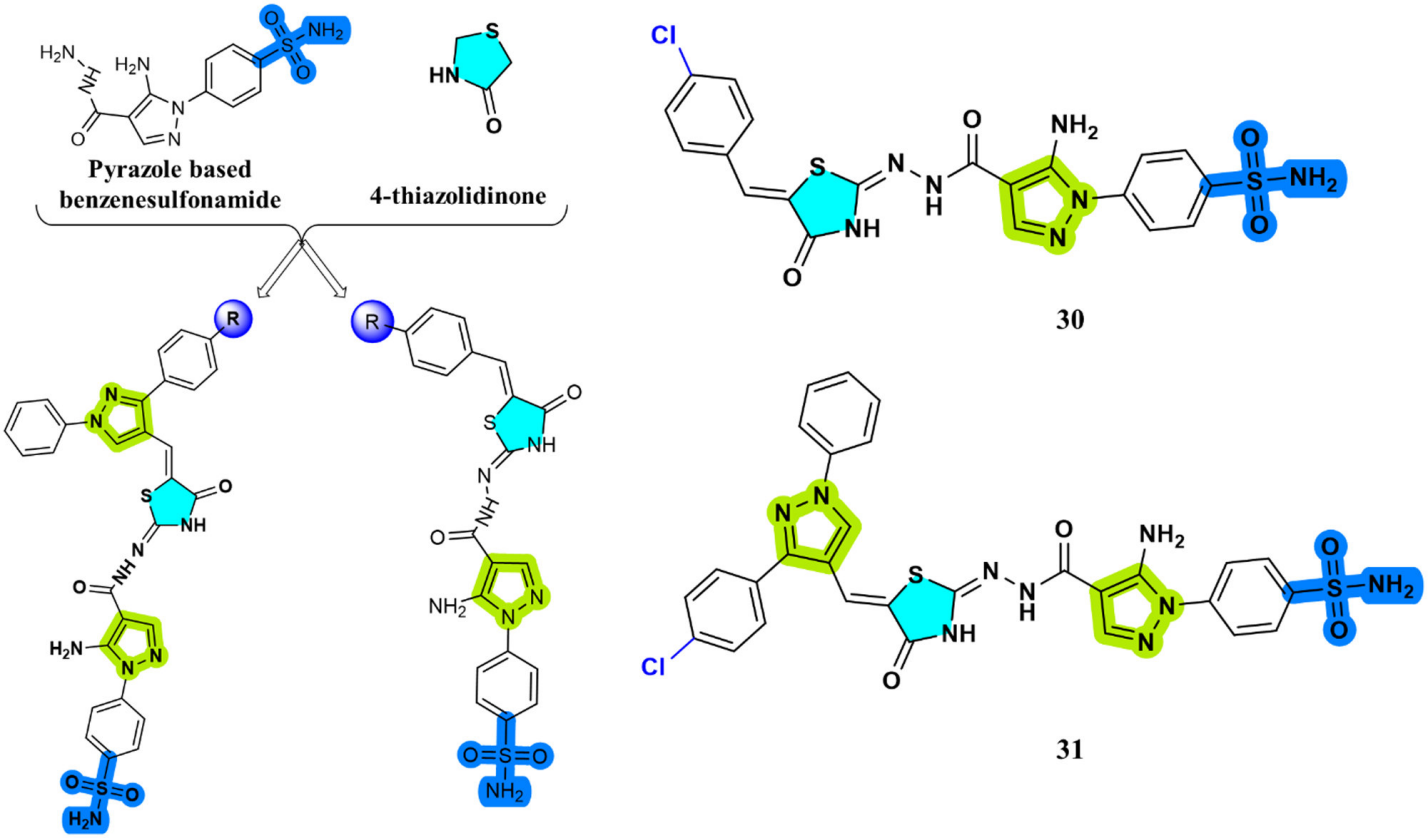
Hybridization strategy of pyrazole‐containing sulfonamides and 4‐thiazolidinones, showing the design approach and derivatives as anti‐inflammatory action.

Bari et al. synthesized a series of 4‐thiazolidinone derivatives containing a benzenesulfonamide moiety and evaluated their anti‐inflammatory activity (Figure 15) [98]. Among the synthesized derivatives, compound 32 showed the highest COX‐2 inhibitory activity, producing 61.75% inhibition of COX‐2 and only 0.35% inhibition of COX‐1 at 1 µM, with a reported selectivity index of 176.42. In comparison, celecoxib showed 75.12% COX‐2 inhibition, 0.45% COX‐1 inhibition, and a selectivity index of 166.93. Thus, although compound 32 showed lower COX‐2 inhibition than celecoxib, it displayed a slightly higher COX‐2 selectivity index. In the carrageenan‐induced rat paw edema assay, compound 32 produced 74.44, 79.49, and 88.75% inhibition at 1, 3, and 5 h, respectively, at a dose of 20 mg/kg. Under the same conditions, celecoxib produced 92.17, 100, and 100% inhibition, respectively. Therefore, compound 32 was the most active derivative of the synthesized series, although its in vivo anti‐inflammatory effect remained lower than that of celecoxib.

**FIGURE 15:**
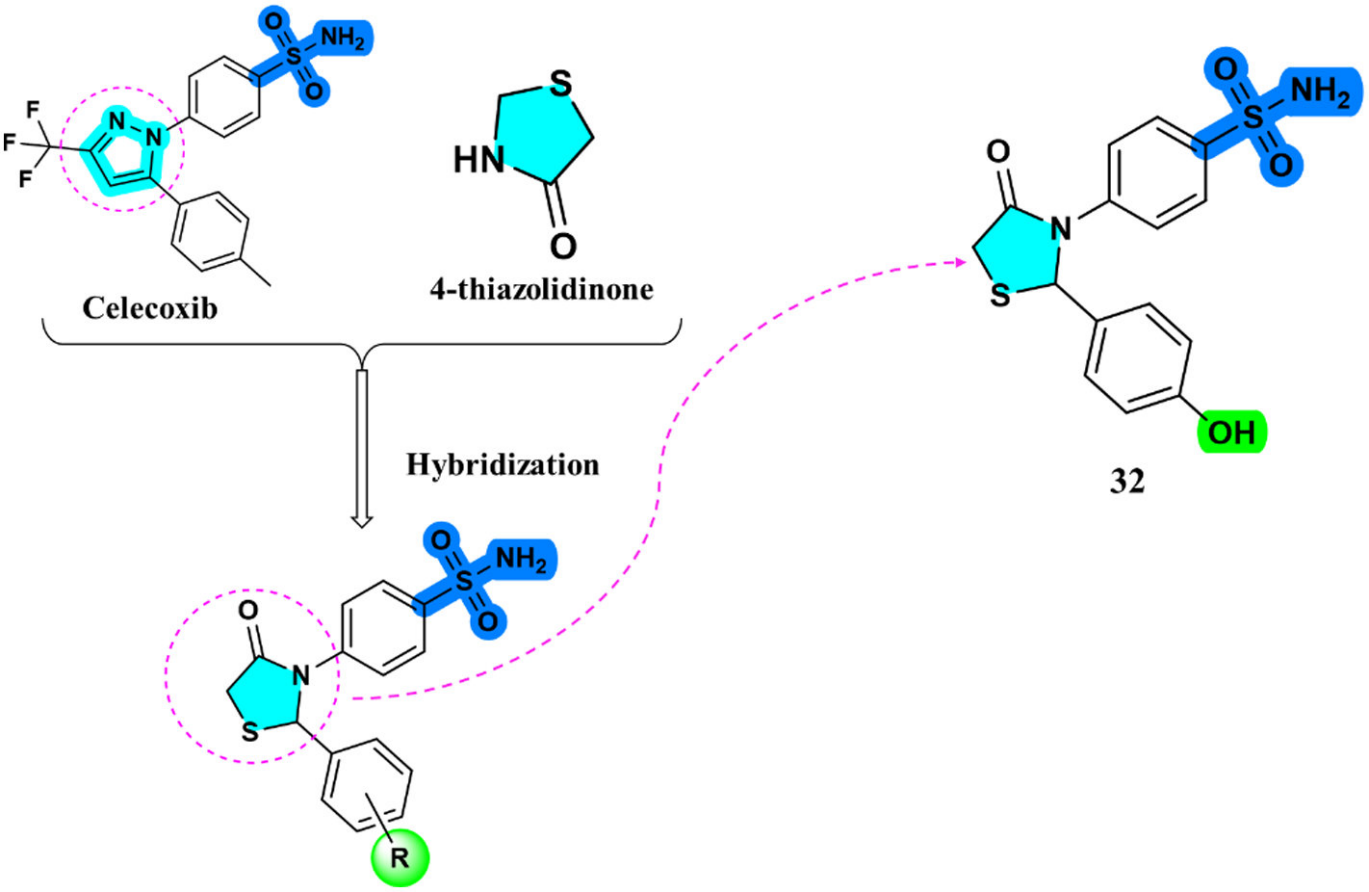
Design and structures of benzenesulfonamide‐4‐thiazolidinone hybrids developed as selective COX‐2 inhibitors.

Abdellatif et al. synthesized novel series of 4‐thiazolidinones containing sulfonamide (Figure 16) [99]. Among the synthesized compounds, 33 and 34 showed the most favorable COX‐2 inhibitory and anti‐inflammatory profiles. Compound 33 inhibited COX‐2 with an IC_50_ of 2.3 µM and showed a selectivity index of 4.56, whereas compound 34 exhibited a COX‐2 IC_50_ of 1.9 µM with a selectivity index of 5.68. In comparison, the standard drug celecoxib showed a COX‐2 IC_50_ of 1.33 µM and a higher selectivity index of 7.29. In the carrageenan‐induced paw edema assay at 20 mg/kg, compounds 33 and 34 produced 61.8% and 67.0% inhibition after 3 h and 64.5% and 69.6% after 5 h, respectively, compared with 60.0% and 62.5% inhibition for celecoxib at the corresponding time points. Their ED_50_ values were 27.7 and 18.1 µmol/kg, respectively, compared with 28.2 µmol/kg for celecoxib, indicating particularly favorable in vivo potency for compound 34. Moreover, compounds 33 and 34 showed 85% and 92% reductions in ulcerogenic potential, respectively, relative to celecoxib. Thus, although their in vitro COX‐2 potency and selectivity were lower than those of celecoxib, both derivatives showed comparable in vivo anti‐inflammatory activity with a markedly improved gastric safety profile.

**FIGURE 16:**
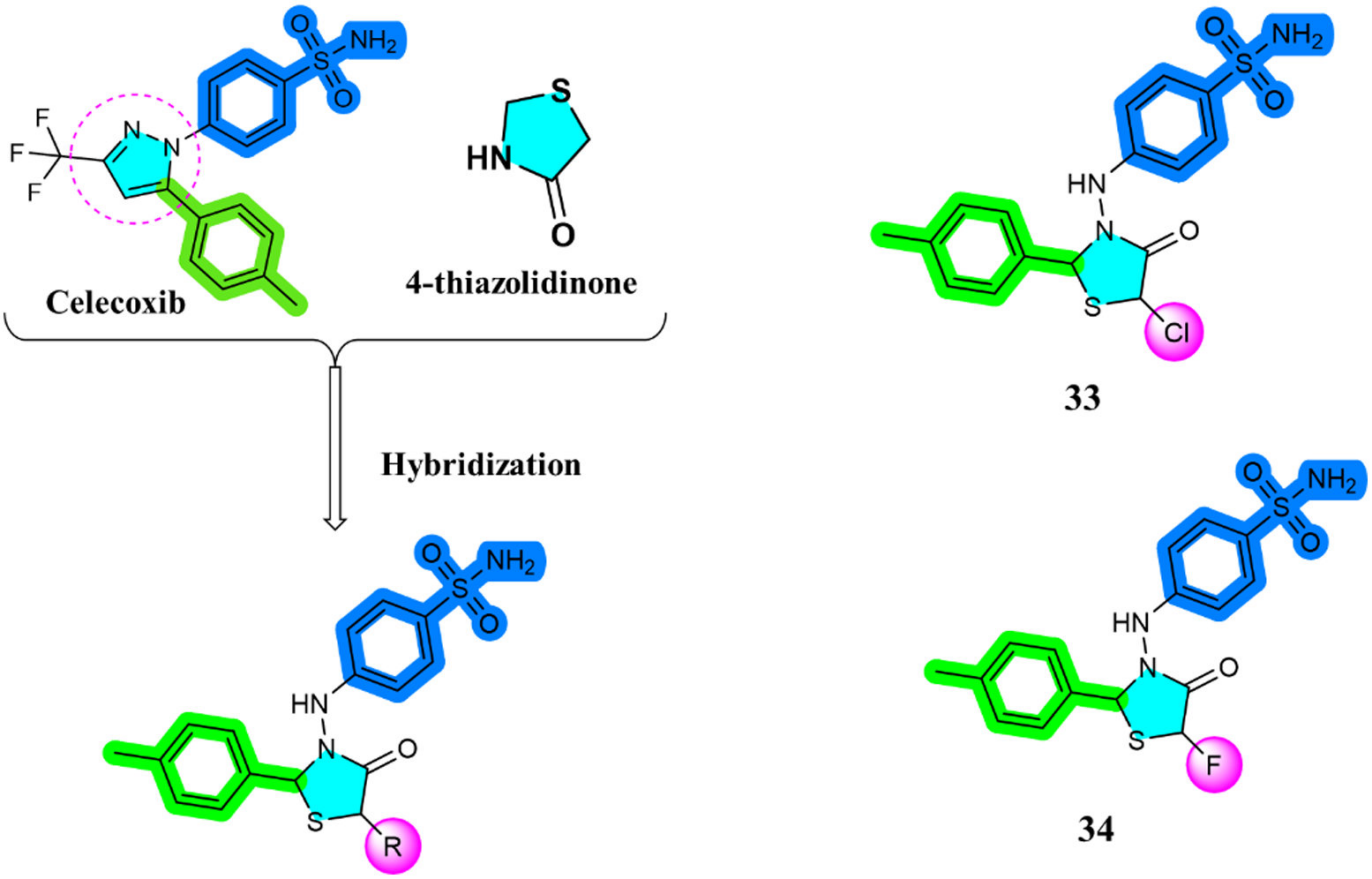
Design and structures of sulfonamide‐containing 4‐thiazolidinone derivatives with anti‐inflammatory activity.

### 3.4 Human Carbonic Anhydrase Inhibitory Activity

Mahmood et al. designed and synthesized a series of iminothiazolidinone–sulfonamide hybrids through heterocyclization of sulfanilamide thioureas with methyl bromoacetate and evaluated them as inhibitors of hCA I, II, IV, and IX (Figure 17) [100]. Among the synthesized derivatives, compounds 35 and 36 showed pronounced inhibitory activity, particularly against hCA II. Compound 36 was the most potent hCA II inhibitor, with a K_i_ value of 0.41 nM, followed by compound 35 (0.53 nM); both were considerably more potent than the reference drug acetazolamide K_i_ value of 12 nM. Against the tumor‐associated hCA IX isoform, compound 35 showed the highest activity, with a K_i_ value of 24.3 nM, which was slightly better than that of acetazolamide (25 nM), whereas compound 36 showed a K_i_ value of 42.1 nM. In contrast, both derivatives showed weaker inhibition of hCA IV, with K_i_ values of 390.1 and 932.8 nM for compounds 35 and 36, respectively, compared with 74 nM for acetazolamide. Thus, compound 36 was the most potent hCA II inhibitor, whereas compound 35 displayed the most favorable activity against hCA IX.

**FIGURE 17:**
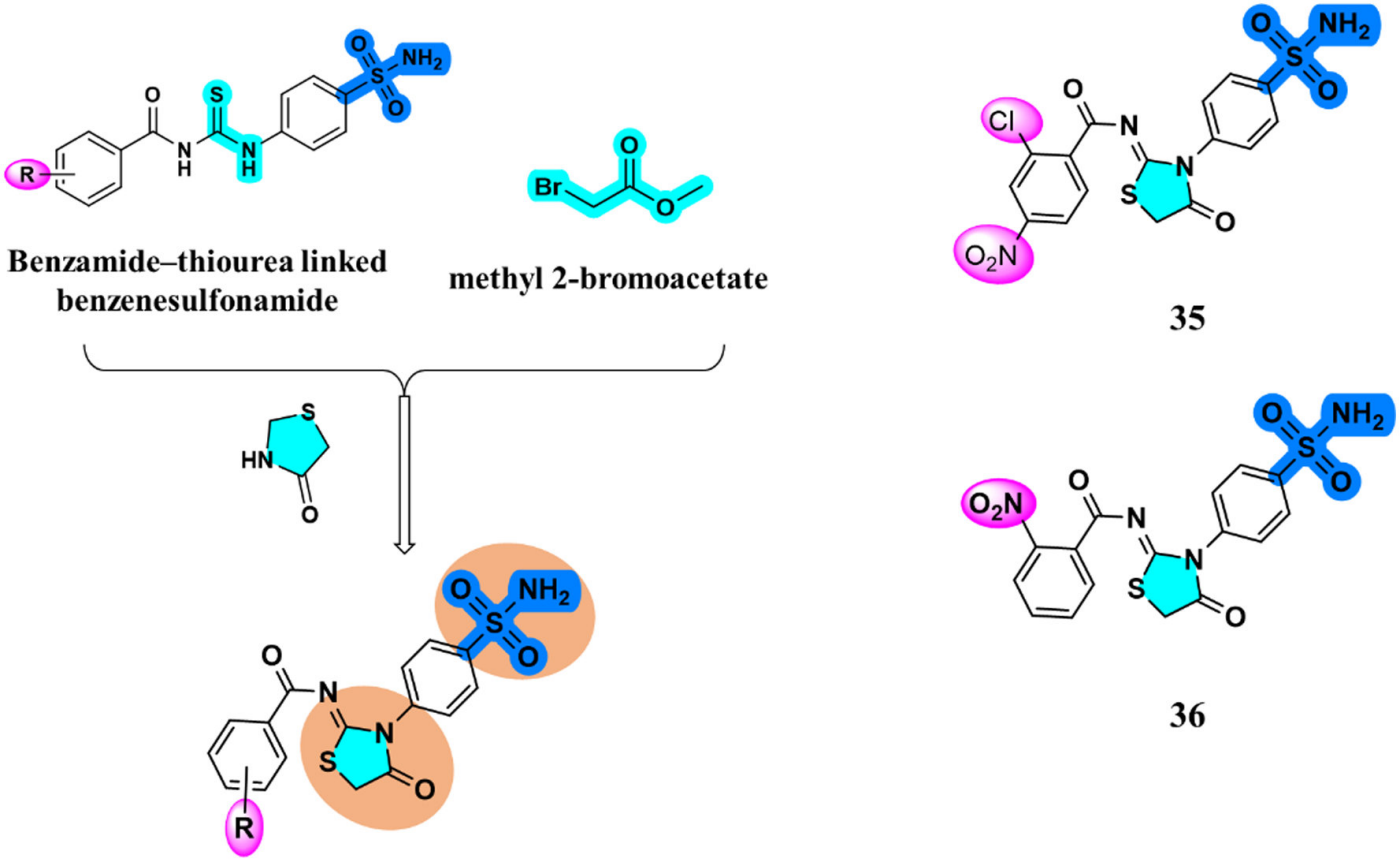
Design strategy of benzamide containing 4‐thiazolidinone–sulfonamide hybrid highlights the transformation of the thiourea linker into a 4‐thiazolidinone ring and derivatives as hCA inhibitors.

Zhang et al. reported a series of benzenesulfonamide–thiazolidinone hybrids and evaluated their inhibitory activity against hCA II and the tumor‐associated hCA IX isoform (Figure 18) [101]. Among the synthesized derivatives, compound 37 showed the most favorable overall activity, with IC_50_ values of 115.4 nM against hCA II and 117.2 nM against hCA IX. Compound 38 also displayed notable inhibition, with IC_50_ values of 121.3 and 146.5 nM against hCA II and hCA IX, respectively, whereas compound 39 showed greater activity toward hCA IX (130.5 nM) than hCA II (146.5 nM). In comparison, the reference inhibitor acetazolamide showed IC_50_ values of 30.47 nM against hCA II and 88.1 nM against hCA IX, indicating higher potency than the synthesized derivatives. However, compounds 37 and 38 were more active than SLC‐0111, which exhibited IC_50_ values of 137.8 and 180.7 nM against hCA II and hCA IX, respectively. Mafenide hydrochloride showed considerably weaker inhibition, with IC_50_ values of 8912 nM against hCA II and >30,000 nM against hCA IX. The substitution pattern markedly influenced inhibitory activity, with methoxy‐containing derivatives, particularly compounds 37 and 38, showing favorable activity, whereas bulky or strongly electron‐withdrawing substituents such as benzyloxy, isopropyl, and trifluoromethyl generally reduced potency.

**FIGURE 18:**
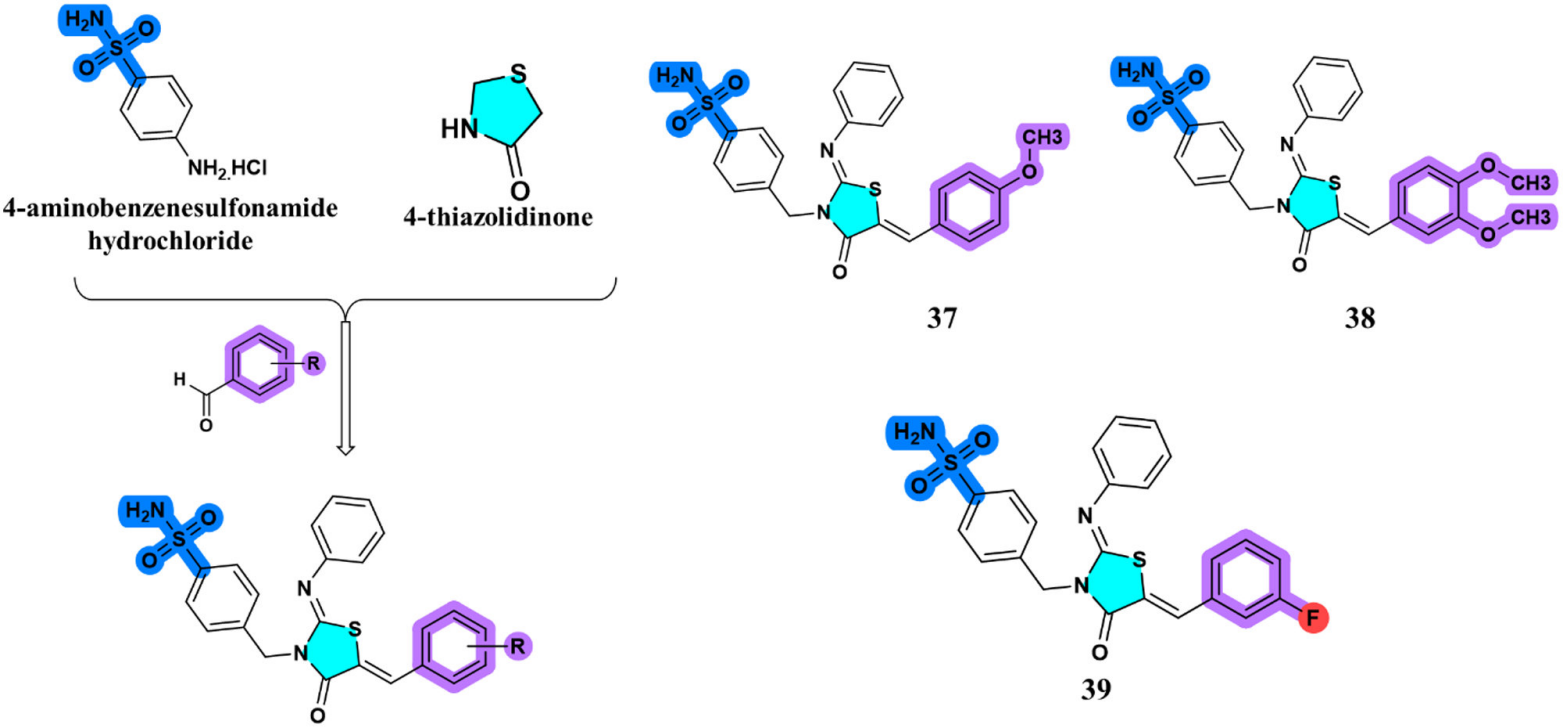
Design of benzenesulfonamide–thiazolidinone hybrids and structures of the most active hCA II/hCA IX inhibitors, derivatives 37–39.

Nemr et al. designed and synthesized a novel series of thiazole‐benzenesulfonamide derivatives (Figure 19) featuring various aryl substitution, primarily as carbonic anhydrase inhibitors, with additional evaluation of their anticancer, antibacterial, and antibiofilm activities [102]. Among the synthesized derivatives, compound 40 showed the strongest inhibition of the tumor‐associated hCA IX isoform, with an IC_50_ value of 0.011 µM, compared with 0.00883 µM for dorzolamide and 0.0045 µM for SLC‐0111. Compound 41 also showed marked hCA IX inhibition (0.017 µM) and exhibited an IC_50_ of 1.55 µM against hCA II, indicating pronounced selectivity toward hCA IX. Compound 42 displayed the highest cytotoxic activity, with IC_50_ values of 1.56 µM against MDA‐MB‐231 and 1.52 µM against MCF‐7 cells, compared with 7.67 and 5.89 µM, respectively, for the reference drug staurosporine. Moreover, 42 showed lower toxicity toward normal MCF‐10A cells (IC_50_ = 26.1 µM) than staurosporine (13.0 µM). In the antibacterial assay, compound 40 produced 80.69% inhibition against *S. aureus*, compared with 99.25% for ciprofloxacin, whereas compounds 41 and 42 showed notable antibiofilm activity against *K. pneumoniae*, with inhibition values of 79.46% and 77.52%, respectively. Overall, compounds 40, 41, and 42 displayed complementary biological profiles, with 40 showing the strongest hCA IX inhibition, 42 the greatest anticancer activity, and 41 notable CA IX‐selective and antibiofilm activities.

**FIGURE 19:**
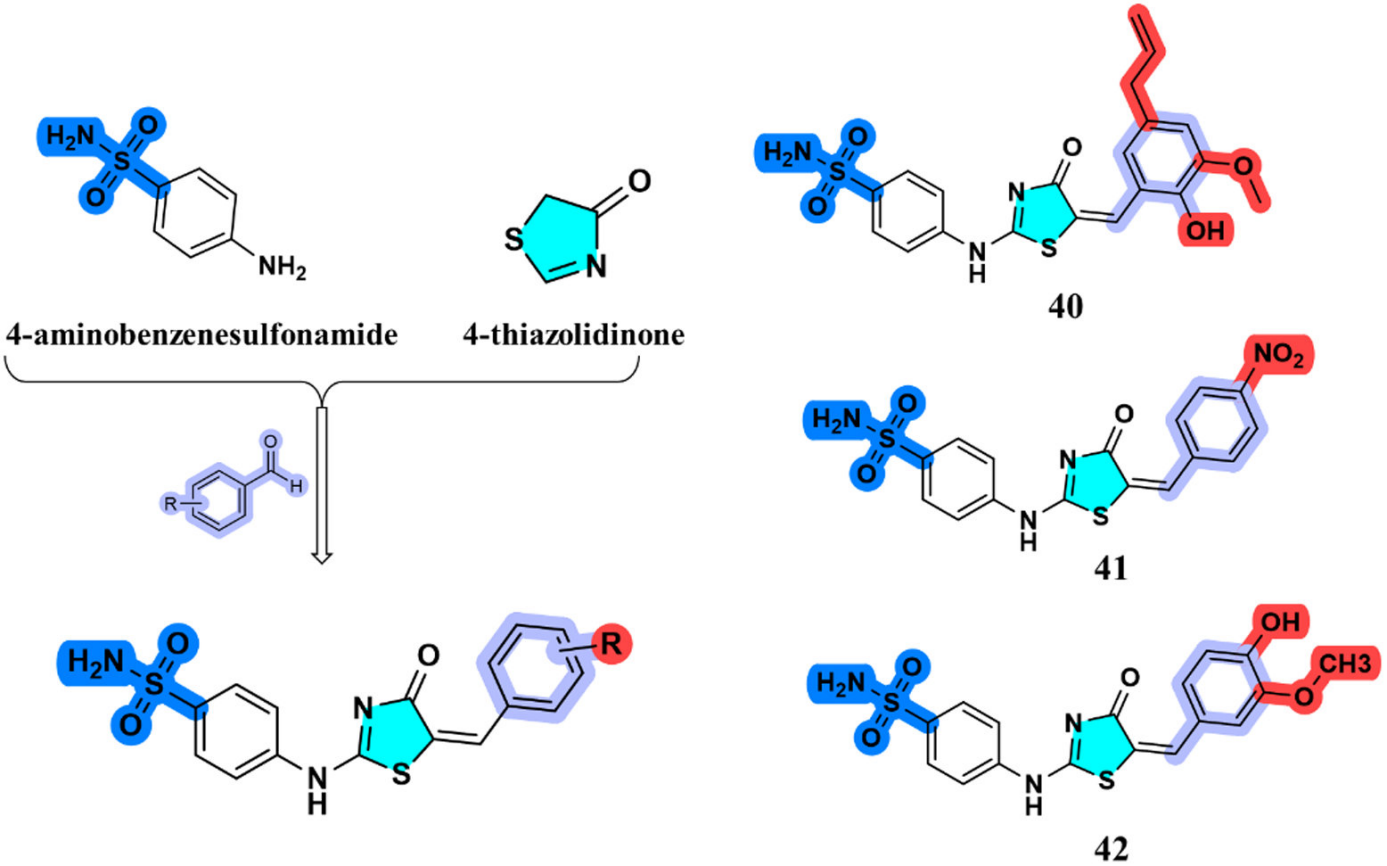
Design strategy for the 4‐thiazolidinone–sulfonamide hybrids with hCA IX inhibitor derivatives.

Onali et al. synthesized a new series of thiazolidinone–isatin hybrids containing a benzenesulfonamide scaffold and the design was based on combining three pharmacologically important scaffolds (Figure 20), thiazolidinone, isatin, and sulfonamide, to obtain selective inhibitors of the tumor‐associated isoforms hCA IX and hCA XII [103]. Compound 43, bearing a fluorine substituent at the 7‐position of the isatin ring, showed the most promising activity, with Ki values of 57.8 nM against hCA IX and 44.3 nM against hCA XII. In comparison, the reference inhibitor acetazolamide exhibited Ki values of 25.8 and 5.7 nM against hCA IX and hCA XII, respectively, indicating greater inhibitory potency. However, compound 43 showed markedly improved selectivity over hCA II, with hCA II/hCA IX and hCA II/hCA XII selectivity evidences of 5.89 and 7.69, respectively, compared with 0.47 and 2.10 for acetazolamide. These findings indicate that the 7‐fluoro substitution favored selectivity toward the tumor‐associated carbonic anhydrase isoforms, identifying compound 43 as a promising scaffold for further optimization of selective hCA IX/XII inhibitors [104].

**FIGURE 20:**
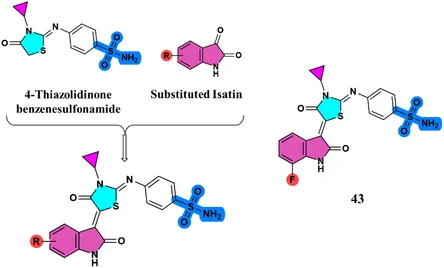
Design of benzenesulfonamide‐containing thiazolidinone–isatin hybrids and structure of the most promising selective hCA IX/XII inhibitor, compound 43.

### 3.5 Antidiabetic Activity

Swapna et al. synthesized a new series of sulfonamide‐conjugated thiazolidine‐2,4‐dione derivatives and evaluated their antidiabetic activity in an alloxan‐induced diabetic model (Figure 21) [105]. Among the synthesized derivatives, compound 44 showed the most pronounced antihyperglycemic activity, reducing blood glucose levels from 343.0 ± 5.797 mg/dL at 0 h to 313.8 ± 9.411 mg/dL at 3 h and 303.2 ± 9.827 mg/dL at 6 h. In comparison, the standard drug metformin reduced blood glucose levels from 343.3 ± 6.206 mg/dL at 0 h to 322.8 ± 4.989 mg/dL at 3 h and 292.0 ± 7.767 mg/dL at 6 h. Compound 44 therefore produced a greater reduction than metformin at 3 h, whereas metformin showed a stronger glucose‐lowering effect at 6 h. Among the synthesized compounds, compound 44 displayed the most sustained antihyperglycemic effect and was identified as the most active derivative of the series.

**FIGURE 21:**
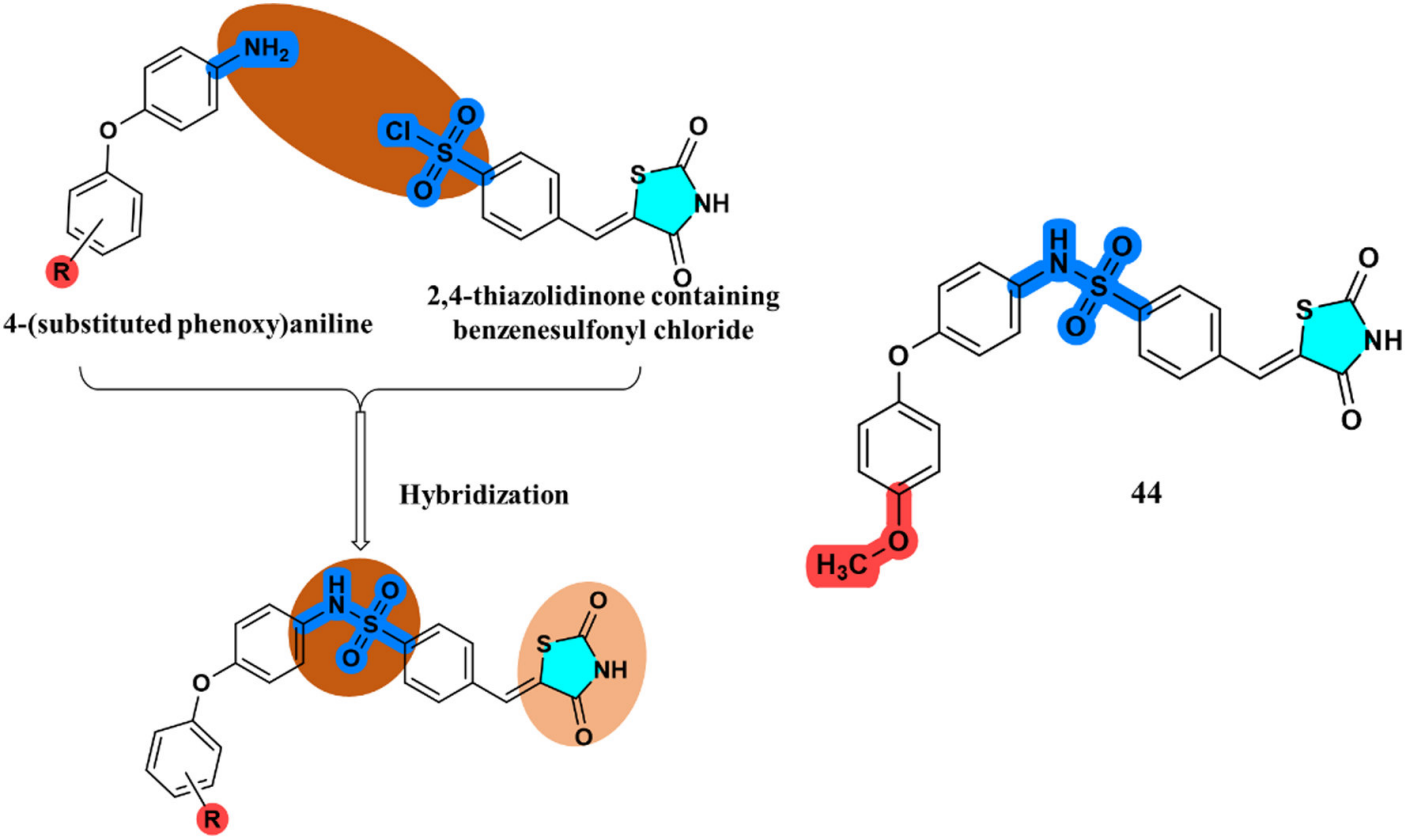
Design of sulfonamide‐conjugated thiazolidine‐2,4‐dione hybrids and most active antidiabetic derivative, compound 44.

Gamal et al. developed a series of benzenesulfonamide–thiazolidinone hybrids (Figure 22) as multitarget‐directed ligands for the management of type 2 diabetes mellitus by simultaneously targeting *α‐glucosidase* and hCA II . The synthesized derivatives showed *α‐glucosidase* inhibitory activity with IC_50_ values ranging from 0.3456 to 3.346 µM. Compound 45 was the most potent *α‐glucosidase* inhibitor, with an IC_50_ value of 0.3456 µM, showing greater activity than the acarbose (IC_50_ = 0.4206 µM) [106]. Compound 46 also showed strong *α‐glucosidase* inhibition (IC_50_ = 0.4400 µM), comparable to acarbose, and displayed the highest hCA II inhibitory activity of the series, with a Ki value of 7.0 nM, exceeding the potency of the reference inhibitor acetazolamide (Ki = 12.0 nM). Thus, compound 46 showed the most favorable dual‐target profile, combining strong *α‐glucosidase* inhibition with potent hCA II inhibition. In the oral glucose tolerance study, compound 46 reduced blood glucose levels to 217 mg/dL, compared with 234 mg/dL for acarbose, further supporting its antihyperglycemic activity. These findings support benzenesulfonamide–thiazolidinone hybrids as a useful scaffold for the development of multitarget antidiabetic agents.

**FIGURE 22:**
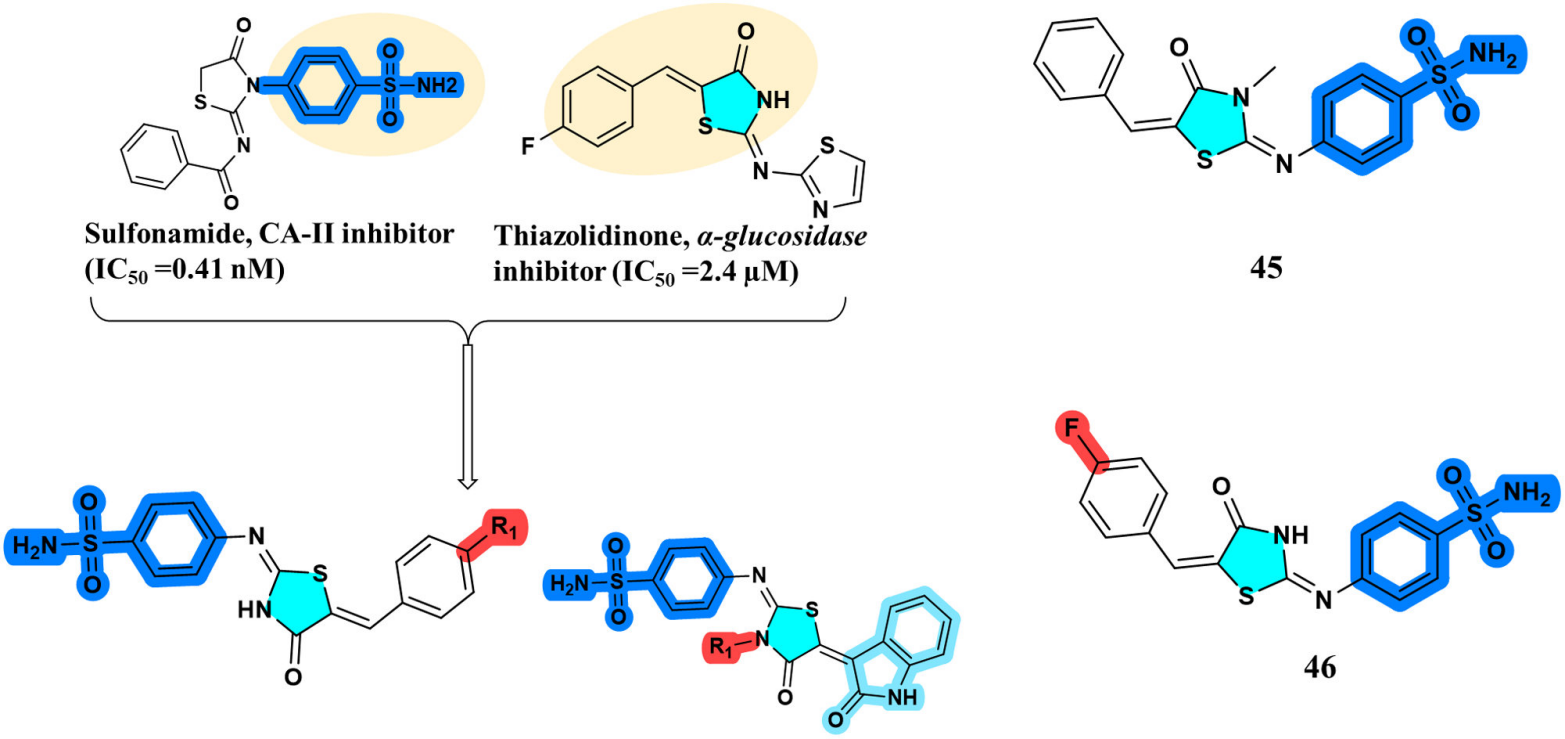
Molecular design strategy and the lead antidiabetic hybrid derivatives compound 45 and 46.

Ranade et al. synthesized a series of benzenesulfonamide–thiazolidin‐4‐one hybrids and assessed their antidiabetic potential by evaluating *α‐amylase* inhibition, glucose uptake, and PPAR‐γ transcription factor activity (Figure 23) [107]. Among all derivatives, compound 47 demonstrated the most favorable activity. Compound 47 inhibited *α‐amylase* with an IC_50_ of 29.51 ± 1.35 µg/mL, which is comparable to the standard drug acarbose (IC_50_ = 26.89 ± 0.116 µg/mL) and also achieved 88.71% glucose uptake at 200 µg/mL, compared with 91.64% for pioglitazone at the same concentration. Furthermore, compound 47 induced a concentration‐dependent increase in PPAR‐γ expression across the tested range of 6.25–100 µM, with pioglitazone serving as the positive control. Overall, these findings highlight compound 47 as the lead derivative in this series, exhibiting *α‐amylase* inhibition, enhanced glucose uptake, and PPAR‐γ modulation.

**FIGURE 23:**
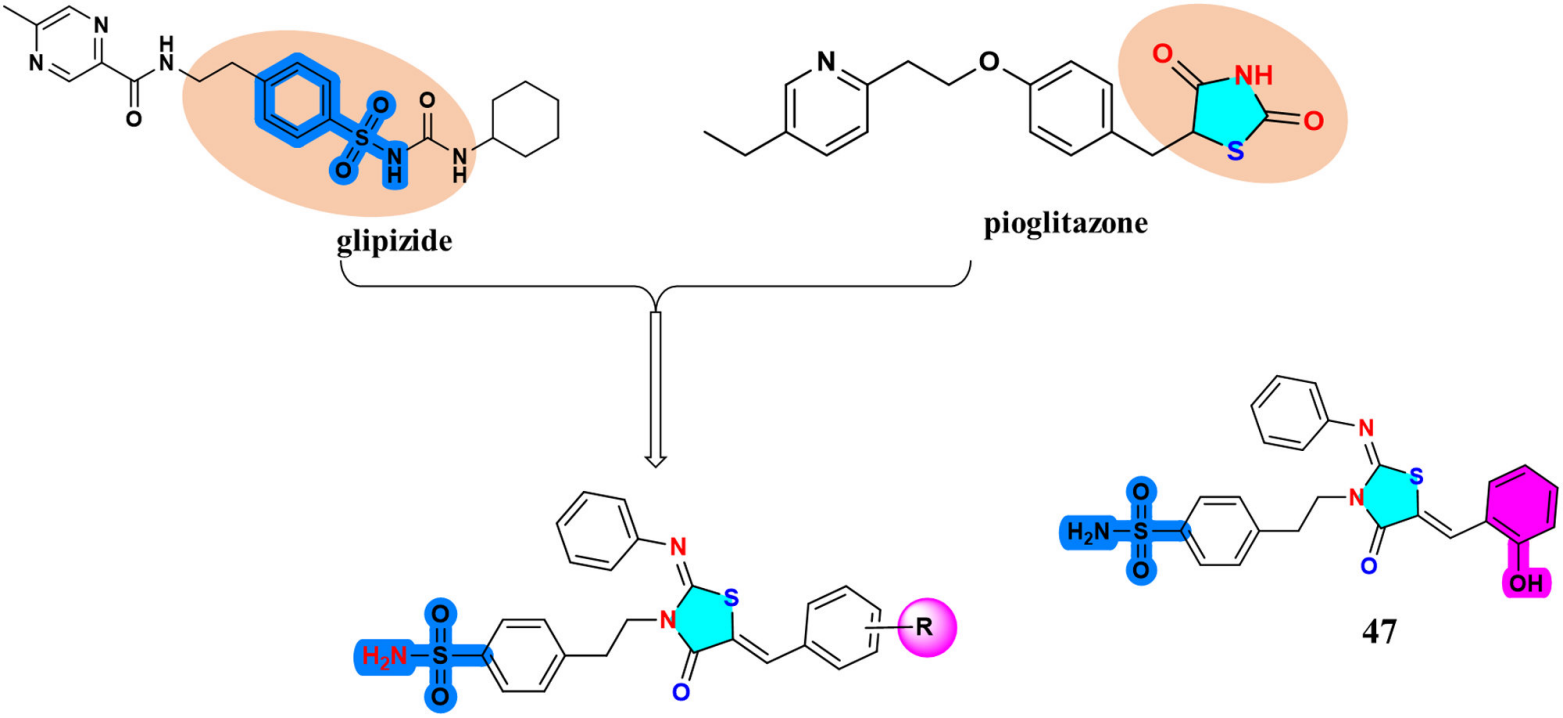
Design of benzenesulfonamide–thiazolidin‐4‐one hybrids and structure of the antidiabetic derivative compound 47.

Ali et al. designed and synthesized a series of thiazolidin‐4‐one‐benzenesulfonamide hybrids as PPARγ agonists and evaluated their antidiabetic activity in both in vitro and in vivo models (Figure 24). Among the synthesized derivatives, compounds 48 and 49 demonstrated the most favorable pharmacological profiles. Compound 49 exhibited the strongest PPARγ agonistic activity (EC_50_ = 2.94 ± 0.09 µM), followed by compound 48 (EC_50_ = 3.98 ± 0.12 µM), although both were less potent than the reference drug pioglitazone (EC_50_ = 0.65 ± 0.01 µM). In NA‐STZ‐induced diabetic rats, compounds 48 and 49 reduced blood glucose levels by 40% and 42%, respectively, compared with a 35% reduction with pioglitazone. Compound 48 also increased serum insulin levels by 4‐fold, compared with a 3.5‐fold increase for pioglitazone, and elevated C‐peptide levels by 48.8%, surpassing the 38.6% increase observed with pioglitazone. Furthermore, compound 48 produced a more pronounced improvement in insulin sensitivity and exerted beneficial effects on hepatic, renal, and oxidative stress parameters. These findings demonstrate compound 48 as the most promising antidiabetic derivative overall, while compound 49 demonstrated the highest PPARγ potency within the synthesized series [108].

**FIGURE 24:**
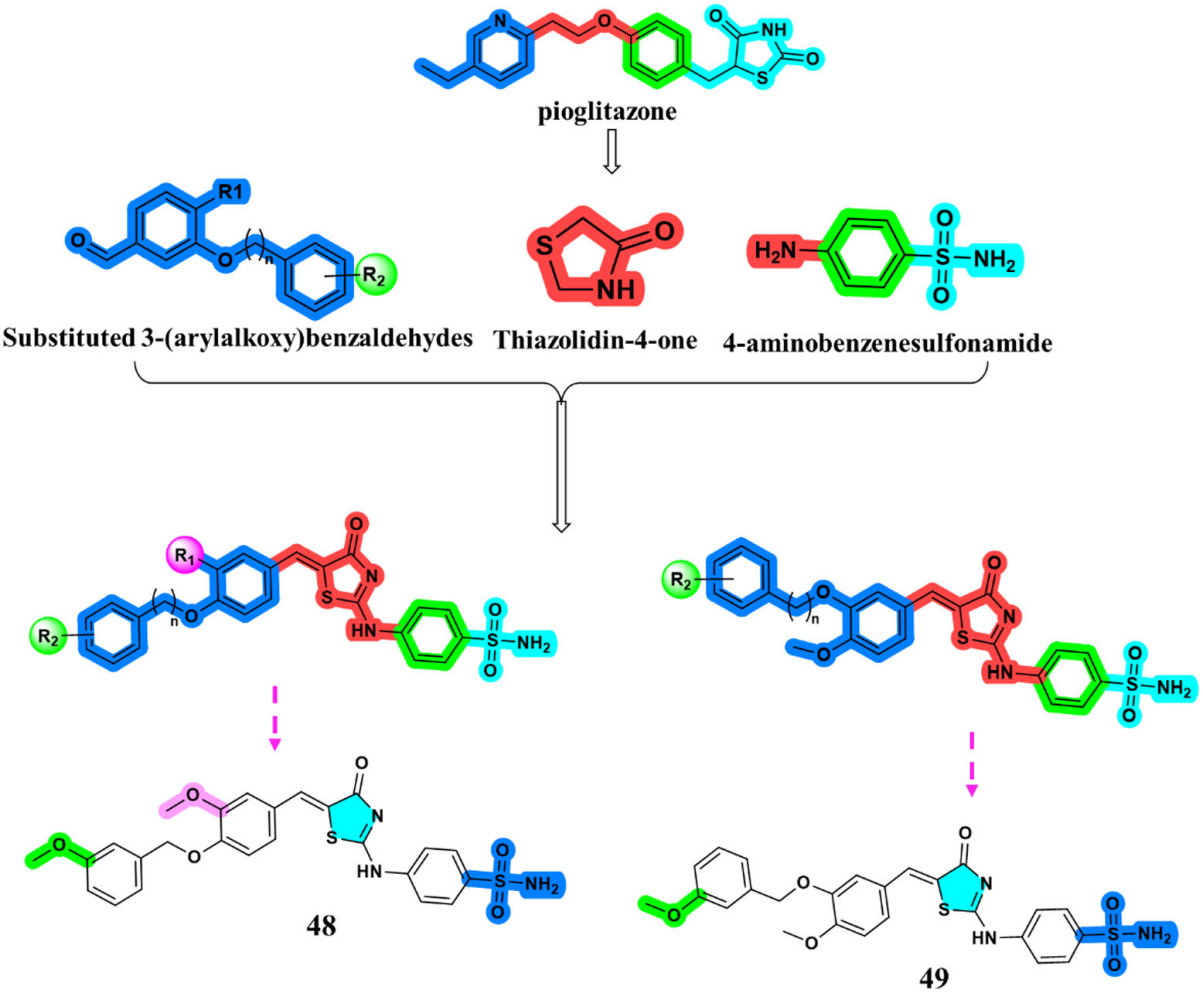
Design of compounds 48 and 49 as PPARγ agonists incorporating thiazolidin‐4‐one and benzenesulfonamide pharmacophores.

### 3.6 Miscellaneous

Siddiqui et al. developed a series of sulfonamide‐containing thiazolidin‐4‐one derivatives and evaluated their anticonvulsant activity using both in vivo and in vitro models (Figure 25) [33]. Among the synthesized derivatives, compounds 50 and 51 demonstrated the highest levels of activity. Compound 50 showed anticonvulsant activity in both the maximal electroshock seizure (MES) and subcutaneous pentylenetetrazole (scPTZ) models, being active at 30 mg/kg in the MES test and at 100 mg/kg in the scPTZ test after 0.5 h, while showing no detectable neurotoxicity up to 300 mg/kg. Compound 51 also showed strong MES activity at 30 mg/kg and exhibited the highest GABA‐T inhibition among the selected derivatives, reaching 30% inhibition at 4 h at 500 µM [109]. In comparison, the standard drug phenytoin was active in the MES test at 30 mg/kg at both 0.5 and 4 h, but was inactive in the scPTZ model at the maximum tested dose and produced neurotoxicity at 100 mg/kg. These results indicate that compound 49 showed the broadest anticonvulsant profile, whereas compound 51 was the most effective GABA‐T inhibitor of the series.

**FIGURE 25:**
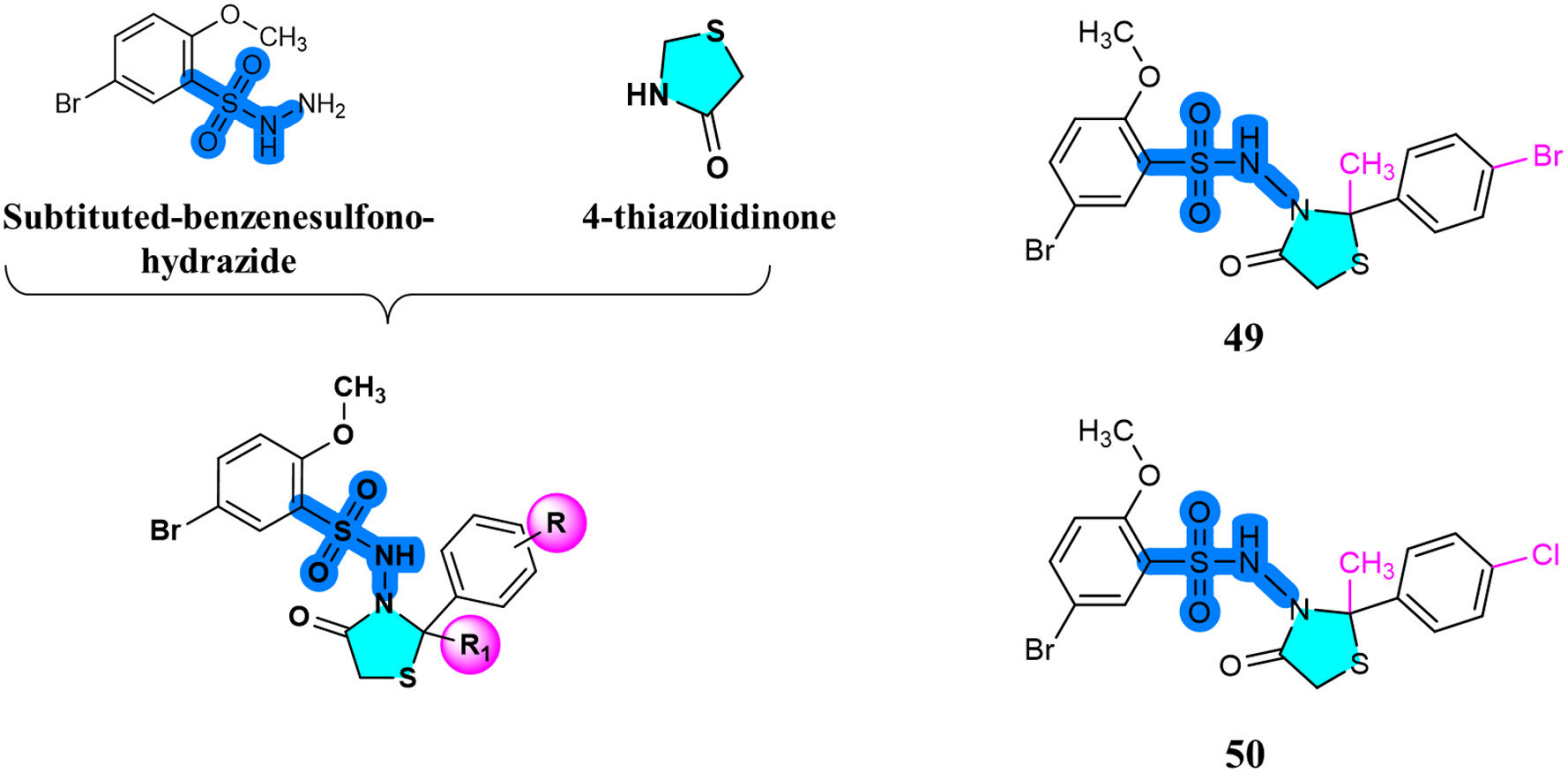
Design of sulfonamide‐containing thiazolidin‐4‐one derivatives and structures of the most promising anticonvulsant compounds.

Preeti and Singh developed a novel one‐pot multicomponent strategy for the synthesis of sulfonamide–thiazolidinone hybrids (Figure 26) using sulfonyl hydrazides, isothiocyanates, and dialkyl acetylene dicarboxylates in ethanol under catalyst‐free conditions at room temperature [110]. The reaction showed excellent efficiency, affording the final products in yields ranging from 79% to 91%, while maintaining high regioselectivity under mild conditions. The methodology was compatible with various aromatic and aliphatic sulfonyl hydrazides as well as substituted isothiocyanates, enabling the preparation of an extensive series of hybrid derivatives [103]. Compound 51, elucidated via single‐crystal X‐ray diffraction, confirmed the exclusive formation of the Z‐configured products (91% yield). Although the pharmacological properties of these compounds were not investigated in this work, the study provides a practical and green synthetic platform for the future development of biologically active sulfonamide–thiazolidinone hybrids.

**FIGURE 26:**
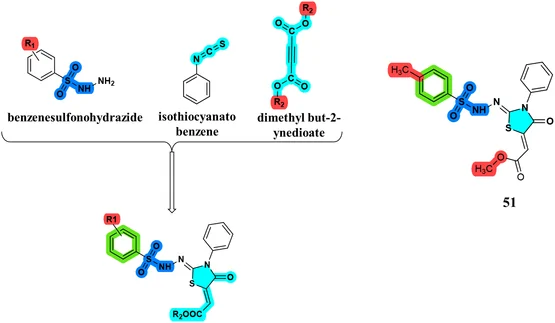
Molecular hybridization design and sulfonamide–thiazolidinone hybrids obtained in high yield.

## 4 Challenges and Limitations

Despite the significant progress in the development of sulfonamide–thiazolidin‐4‐one and sulfonamide–thiazolidine‐2,4‐dione hybrids, several challenges and limitations retain. One of the primary problems is the optimization of pharmacokinetic properties, including bioavailability, metabolic stability, and solubility, which can affect the overall activity of these hybrids in clinical setup [111, 112]. In addition, some hybrids may display off‐target effects or toxicity, required thorough toxicity profile evaluations. Drug resistance, although potentially mitigated by multitarget approaches, remains a concern, particularly in infectious and cancer like diseases [103, 113]. Synthetic complexity and scalability also pose practical limitations, as the development of hybrid molecules with multiple functional groups can be rigorous and costly [114]. Furthermore, limited in vivo studies and clinical trials restrict researchers understanding of the true therapeutic potential and safety profile of these hybrids. Addressing these challenges is essential for translating promising in vitro and in vivo findings into effective clinical applications.

## 5 Future Directions

Future research in the field of sulfonamide–thiazolidin‐4‐one and sulfonamide–thiazolidine‐2,4‐dione hybrids should focus on several key areas. Rational drug design and advanced computational modeling can provide the identification of new hybrid structures with optimal biological and pharmacokinetic profiles. Expanding the scope of biological evaluations, including more comprehensive in vivo studies and early‐stage clinical trials, will be critical for assessing efficacy and safety profile. The incorporation of targeted delivery systems, such as nanoparticles or prodrugs, may enhance bioavailability and reduce off‐target effects. Additionally, exploring new chemical linkers and varying the substitution patterns on the core scaffolds can yield hybrids with improved multitarget activity and selectivity.

## 6 Conclusion

The research on sulfonamide–thiazolidin‐4‐one and sulfonamide–thiazolidine‐2,4‐dione hybrids has opened new possibilities in the area of medicinal chemistry. These hybrid molecules have demonstrated a wide range of biological activities, including antibacterial, antifungal, anticancer, anti‐inflammatory, antidiabetic, and anticonvulsant effects. By merging the strengths of sulfonamides with thiazolidin‐4‐one and thiazolidine‐2,4‐dione scaffolds, scientists have created compounds that can act on multiple targets, which is especially important for treating diseases involving complex or overlapping pathways. The studies reviewed show that careful design and structural modifications can significantly enhance the activity and selectivity of these hybrids. Although challenges remain in optimizing their safety and effectiveness, the promising results so far suggest that sulfonamide–thiazolidin‐4‐one and sulfonamide–thiazolidine‐2,4‐dione hybrids have strong potential as future therapeutic agents. Continued research and development in this area could lead to more effective treatments for a variety of challenging diseases.

## Author Contributions

**Subham Kumar Vishwakarma:** writing – original draft, review and editing, validation, investigation, visualization, conceptualization. **Christian S. Carnero Canales**: writing – original draft. **Sukhwant Singh:** writing – original draft. **Andressa Francielli Bonjorno:** writing – original draft, investigation. **Jean Leandro dos Santos:** writing – review and editing, visualization. **Fernando Rogério Pavan and Cesar Augusto Roque‐Borda:** visualization, validation, supervision, resources, project administration.

## Funding

This study was supported by the National Council for Scientific and this study was financed in part by the Coordenação de Aperfeiçoamento de Pessoal de Nível Superior – Finance code 001/Brasil (CAPES) and the Fundação de Amparo à Pesquisa do Estado de São Paulo (2024/23710‐8, 2025/27766‐0 and 2025/27151‐6).

## Consent

The authors have nothing to report.

## Conflicts of Interest

The authors declare no conflicts of interest.

## Data Availability

No new data were created or analyzed in this study. Data sharing is not applicable to this article.
